# Hypermethylation in CpG-rich cfDNA fragments enables accurate detection of circulating tumor DNA in *BRCA1*- and *BRCA2*-associated breast and ovarian cancer

**DOI:** 10.1038/s41523-026-01076-y

**Published:** 2026-11-11

**Authors:** Aishwarya Sekar, Julian Broche, Veronika Bursic, André Koch, Miguel Camarena Sainz, Jakob Admard, Andreas D. Hartkopf, Ines Gruber, Kristin Bosse, Stephan Ossowski, Christopher Schroeder

**Affiliations:** 1 Institute of Medical Genetics and Applied Genomics, University of Tübingen, Tübingen, Germany; 2 Research Institute for Women’s Health, University of Tübingen, Tübingen, Germany; 3 Department of Women’s Health, University Hospital Tübingen, Tübingen, Germany; 4 Institute for Bioinformatics and Medical Informatics (IBMI), University of Tübingen, Tübingen, Germany

**Keywords:** Biomarkers, Cancer, Genetics, Oncology

## Abstract

Pathogenic *BRCA1* and *BRCA2* germline variants confer an increased risk of hereditary breast and ovarian cancer (HBOC). In this high-risk cohort, novel biomarkers are needed to support patient management. While circulating cell-free DNA-based conventional CpG-level averaging is promising, it dilutes tumor-specific signals in samples with low circulating tumor DNA (ctDNA) fractions. Here, we developed a targeted ultra-deep NGS assay for detecting tumor-derived ctDNA based on fragment-level hypermethylation of CpG-rich cfDNA. A panel of 360 cancer-specific CpG-rich regions derived from 450k methylation array datasets from TCGA breast cancer and GEO ovarian cancer, was applied to plasma from 31 breast cancer, 16 ovarian cancer patients, and 26 healthy controls using enzymatic methylation sequencing. Hypermethylated ctDNA (hm-ctDNA) fragments, were significantly enriched in cancer cases, identifying 62.5% of ovarian and 38.7% of breast cancers at 100% specificity. Elevated hm-ctDNA was observed across different tumor stages, with higher burden in *BRCA1*-associated cases. This approach enables highly specific, non-invasive hypermethylated ctDNA detection in HBOC populations.

## Introduction

*BRCA1* and *BRCA2* are well-established tumor suppressor genes that play a pivotal role in maintaining genomic integrity through DNA damage repair^1^. Pathogenic germline variants in these genes substantially increase the lifetime risk of breast cancer (BC) and ovarian cancer (OC), collectively referred to as hereditary breast and ovarian cancer (HBOC), and represent a major clinical challenge for cancer prevention and early detection^2^. By the age of 80, the cumulative risk of BC is estimated at 72% for *BRCA1* and 69% for *BRCA2* carriers, while the risk of OC reaches 44% and 17%, respectively. In addition, the 20-year cumulative risk of contralateral BC remains high in mutation carriers (40% for *BRCA1* and 26% for *BRCA2*)^3^.

Despite advances in imaging-based surveillance, current screening strategies show limited sensitivity and specificity, particularly for early-stage disease^4,5^. While structured screening programs enable earlier detection of BC^6^, effective screening options for OC are lacking, resulting in most cases being diagnosed at advanced stages and contributing to poor clinical outcomes^7^. In high-risk surveillance programs, a proportion of cancers in *BRCA1*/*2* carriers are still detected at advanced stages^8^. However, even high-grade (III) invasive breast cancers (BC) show limited detection sensitivity (~26% for small lesions < 15 mm), largely due to their non-specific mammographic features compared with lower-grade lesions^9^. This underscores the need for improved technologies capable of detecting small but aggressive tumors. In parallel, accurately distinguishing late-stage ovarian cancer (OC) from breast cancer (BC) ovarian metastases remains both challenging and clinically critical, as management strategies differ substantially^10^. Collectively, these challenges emphasize the strong need for minimally invasive approaches in addition to existing methods to enhance detection and classification of both early- and advanced-stage malignancies, particularly in high-risk populations.

Liquid biopsy approaches based on circulating cell-free DNA (cfDNA) offer a promising, non-invasive strategy to detect tumor-associated molecular alterations. Circulating tumor DNA (ctDNA), the tumor-derived fraction of cfDNA, reflects the genetic landscape of primary and metastatic lesions^11^. However, mutation-based detection of ctDNA remains challenging due to its often-low abundance in plasma, particularly in early-stage disease^12^. Epigenetic alterations, in particular DNA methylation changes, represent an attractive alternative class of biomarkers. Aberrant DNA methylation occurs early during tumorigenesis, is highly tissue-specific, and can therefore improve sensitivity for cancer detection^13^. In the context of *BRCA1* and *BRCA2*-associated cancers, distinct methylation alterations have been reported and may provide additional diagnostic information beyond genetic variants alone^14^. Importantly, cfDNA methylation profiling captures these tumor-associated epigenetic signals in a minimally invasive manner and enables repeated sampling over time^15^.

Beyond sequence and methylation information, cfDNA also carries structural features that reflect its cellular origin. Fragmentation patterns are influenced by nucleosome positioning and chromatin accessibility, resulting in characteristic fragment length distributions^16,17^. While non-tumor cfDNA fragments typically peak around ~167 bp, ctDNA fragments are often shorter (< 145 bp)^18^. Emerging evidence further suggests that DNA methylation influences nuclease cleavage and fragmentation patterns, linking epigenetic state to fragment structure^19^. In addition, specific nucleotide motifs at fragment ends (end motifs) have been reported to differ between tumor-derived and non-tumor cfDNA^20^. However, the relationship between DNA methylation, fragment structure, and ctDNA detection in *BRCA1*- and *BRCA2*-associated cancers remains insufficiently understood.

Most current cfDNA methylation approaches rely on averaging methylation levels across individual CpG sites or genomic regions. This strategy may dilute tumor-specific signals, particularly in samples with low ctDNA fractions. Analyzing methylation at the level of individual cfDNA fragments, by jointly considering multiple CpGs per fragment, has been shown to enhance the detection of tumor-derived cfDNA^21^. In this study, we developed a targeted ultra-deep NGS assay for detection of hypermethylated CpG-rich fragments that identifies tumor-derived ctDNA based on fragment-level hypermethylation patterns affecting at least six CpG sites. We designed a panel of CpG-rich regions showing cancer-specific hypermethylation in TCGA-derived breast and ovarian tumor datasets and applied enzymatic methylation sequencing to cfDNA from blood plasma. By jointly evaluating CpG density and methylation proportion per fragment, we define a class of hypermethylated cfDNA fragments (hm-ctDNA) that are highly enriched in a subset of cancer patients. Our statistical analysis strategy provides robust and easy to interpret results for clinical applications (fraction of hm-ctDNA, fold-changes compared to healthy controls, *P*-values). We demonstrate that our approach enables the detection of ctDNA in plasma of cancer patients with *BRCA1*- and *BRCA2*-associated breast and ovarian cancer with high specificity.

## Results

### Clinical characteristics of the cohort

**Fig. 1:**
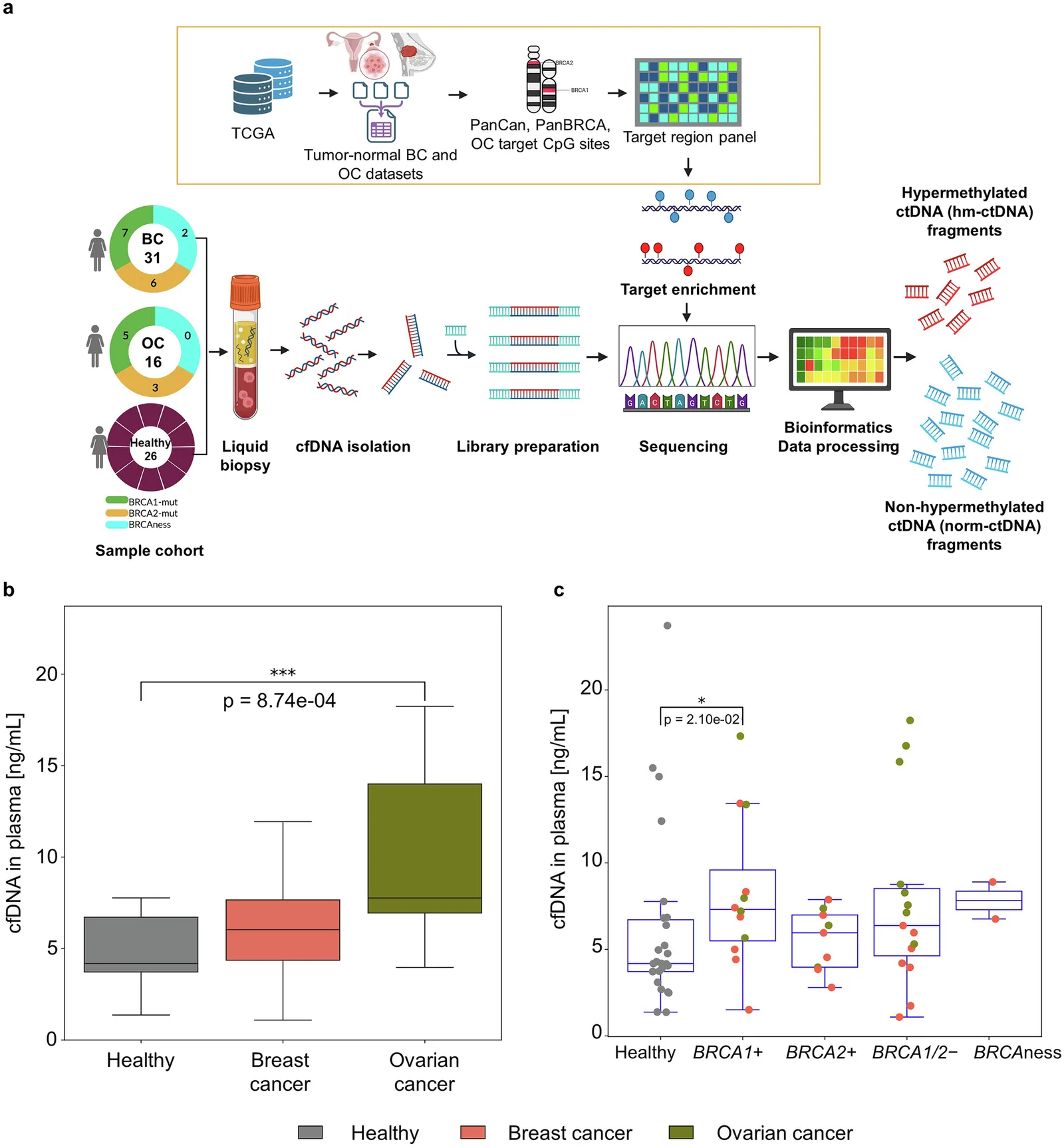
Study overview and cfDNA concentration in plasma. **a** Schematic overview of the study concept and workflow, including cohort selection, sample processing, and downstream cfDNA methylation and fragmentomic analysis. **b** Boxplots showing the cfDNA concentration in plasma **c** Boxplots showing cfDNA concentration in plasma with respect to *BRCA1* and *BRCA2* variant status.

The study was a targeted methylation approach aimed at detecting hypermethylated ctDNA. An overview of the study design and analytical workflow is illustrated in Fig. 1a. The study included 73 individuals: 31 patients with breast cancer (BC), 16 with ovarian cancer (OC), and 26 healthy controls. Clinical characteristics, including tumor stage and grading, are summarized in Table 1. Mean cfDNA concentrations were 4.19 ng/mL (range 1.37–23.72) in healthy individuals, 6.04 ng/mL (1.09–14.45) in BC, and 7.77 ng/mL (3.97–18.24) in OC (Fig. 1b). Among BC patients, 7 carried (likely) pathogenic *BRCA1* germline variants and 6 carried (likely) pathogenic *BRCA2* germline variants, while 2 cases were classified as BRCAness. In OC, 5 patients carried (likely) pathogenic *BRCA1* and 3 carried (likely) pathogenic *BRCA2* germline variants. cfDNA concentration was significantly increased in OC (Fig. 1b) and in *BRCA1*+ (Fig. 1c) patients as compared to healthy controls.

**Table. 1:** Clinical characteristics of the samples

Clinical characteristics	Breast cancer *n* = 31		Ovarian cancer *n* = 16	
Mean cfDNA concentration (ng/ml)	1.09–14.45		3.97–18.24	
(likely) pathogenic *BRCA1* germline variant	7	7/22 31%	5	5/16 31%
(likely) pathogenic germline *BRCA*2 variant	6	6/22 27%	3	3/16 18%
BRCAness	2	2/22 9%	0	0/16 0%
*BRCA1/2* negative	7	7/22 31%	8	8/16 50%
Missing information	9	-	-	-
Grading - G2	12	12/29 41%	0	0/16 0%
G3	17	17/29 58%	16	0/16 100%
Missing information	2	-	0	-
Pathologic tumor Status - T1	0	0/29 0%	2	2/16 12%
T2	8	8/29 27%	1	1/16 6%
T3	18	18/29 62%	9	9/16 56%
T4	3	3/29 10%	0	0/16 0%
Missing information	2	-	-	-

### Panel design and genomic annotation of included regions

Using primary tumor data of cases from various publicly available BC and OC databases such as TCGA, we identified 360 regions with at least one hypermethylated CpG in BC or OC, which were used for NGS panel design (see Methods). CpGs significant in both OC and BC were classified as pan-cancer. The genomic context of the 360 panel regions was characterized based on Ensembl, including overlap to genes, TSS, regulatory regions such as promoters and enhancers, and CpG islands (Supplementary Table 2 and Supplementary Fig. 2). Several genomic regions contained multiple closely clustered hypermethylated CpGs, suggesting hotspots of methylation changes. Notable multi-CpG loci included *HSPA1A* (12 CpGs), *ZIC1* (6 CpGs), *CDKL2* (8 CpGs), *UBB* (5 CpGs), *ZNF69* (6 CpGs), *CLDN10* (5 CpGs), *SOX1/SOX1-OT*, and *AC002472.1* (6 CpGs). The majority of CpGs were located within CpG islands, shores, and shelves. A large fraction of regions overlaps promoters/TSS, followed by CTCF binding sites and enhancers. Based on the overlap with promoters/TSS or gene bodies most regions can be associated to a gene.

### Fragmentomic features of hypermethylated ctDNA

**Fig. 2:**
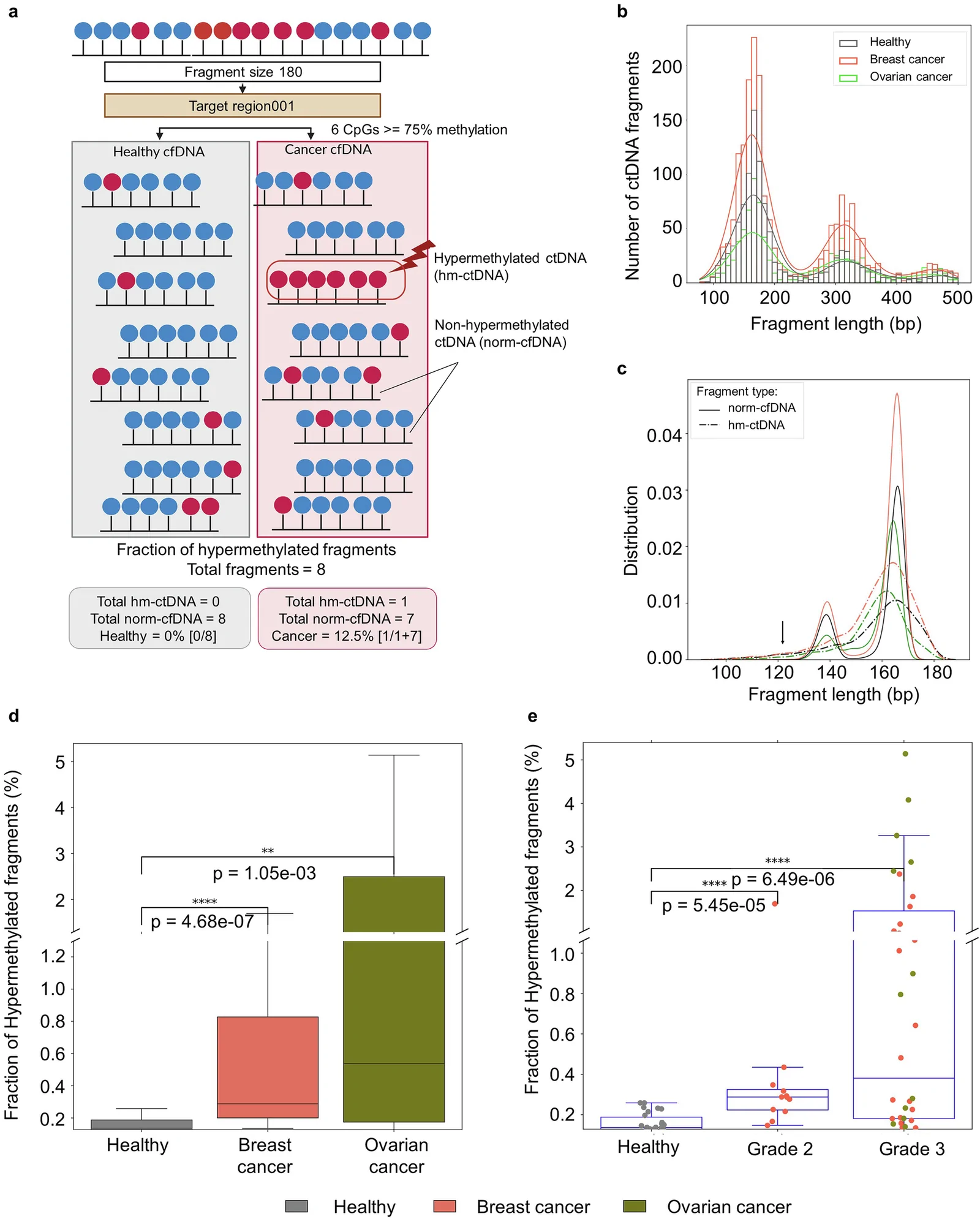
Detection of hypermethylated fragments. **a** Concept of distinguishing hypermethylated ctDNA (hm-ctDNA) from non-hypermethylated cfDNA. **b** Fragment size distribution and counts of ctDNA fragments identified in all the samples. **c** Fragment size distribution of long (125-180) hypermethylated ctDNA fragments. **d** Percentage of hm-ctDNA fractions across groups. The median of healthy controls was 0.14% with an IQR of 0.12% to 0.19%. BC had a median of 0.29% and IQR of 0.20% to 0.83%. OC had a median of 0.54% with an IQR of 0.17% to 2.50%. Due to the smaller OC group size compared to BC (16 vs 31), the *p*-value is less significant, despite the higher median. **e** Percentage of hm-ctDNA fractions with target regions at different grading.

To detect tumor-derived cfDNA, we quantified fragment-level hypermethylation by distinguishing hypermethylated ctDNA (hm-ctDNA) as fragments containing ≥6 CpGs with ≥75% methylated CpGs, and normal cfDNA (norm-cfDNA) as fragments containing ≥6 CpGs with < 75% methylated CpGs (the concept is depicted in Fig. 2a). Hm-ctDNA load was then calculated as ratio of hm-ctDNA to all fragments with ≥6 CpGs (see Methods). To assess whether hm-ctDNA exhibits distinct structural features, we analyzed fragment length distributions and end-motif patterns. For all ctDNA combined we observed the expected pattern of fragment lengths defined by histone circumference, with peaks at 160 bp, 320 bp, and 480 bp (Fig. 2b). hm-ctDNA fragments showed a dominant peak at ~165 bp and lacked the shorter fragment peak (~145 bp) observed in norm-cfDNA (Fig. 2c), suggesting an association between hypermethylation and nucleosome-protected DNA fragments.

### Fragment-level hypermethylation is enriched in breast and ovarian cancer-derived cfDNA

Across the cohort, hm-ctDNA load was significantly increased in both BC and OC samples compared to healthy controls (Fig. 2d). Differences were highly significant for BC (*P* = 4.68 × 10⁻⁷) and OC (*P* = 1.05 × 10⁻³). Stratification by clinical parameters revealed that hm-ctDNA load was elevated across tumor grades and stages. In BC, both grade 2 and grade 3 tumors showed significantly higher hm-ctDNA load compared to controls (*P* = 5.45 × 10⁻⁵ and *P* = 6.49 × 10⁻⁶, respectively; Fig. 2e). Similarly, increased hm-ctDNA load was observed for all tumor stages (Supplementary Fig. 3a) and HER2 subtypes (Supplementary Fig. 3b).

These results demonstrate that fragment-level hypermethylation is consistently enriched in cfDNA from cancer patients within our panel’s target regions and reflects disease burden across clinical subgroups.

### Ovarian cancer samples show distinct fragment-level hypermethylation patterns

**Fig. 3:**
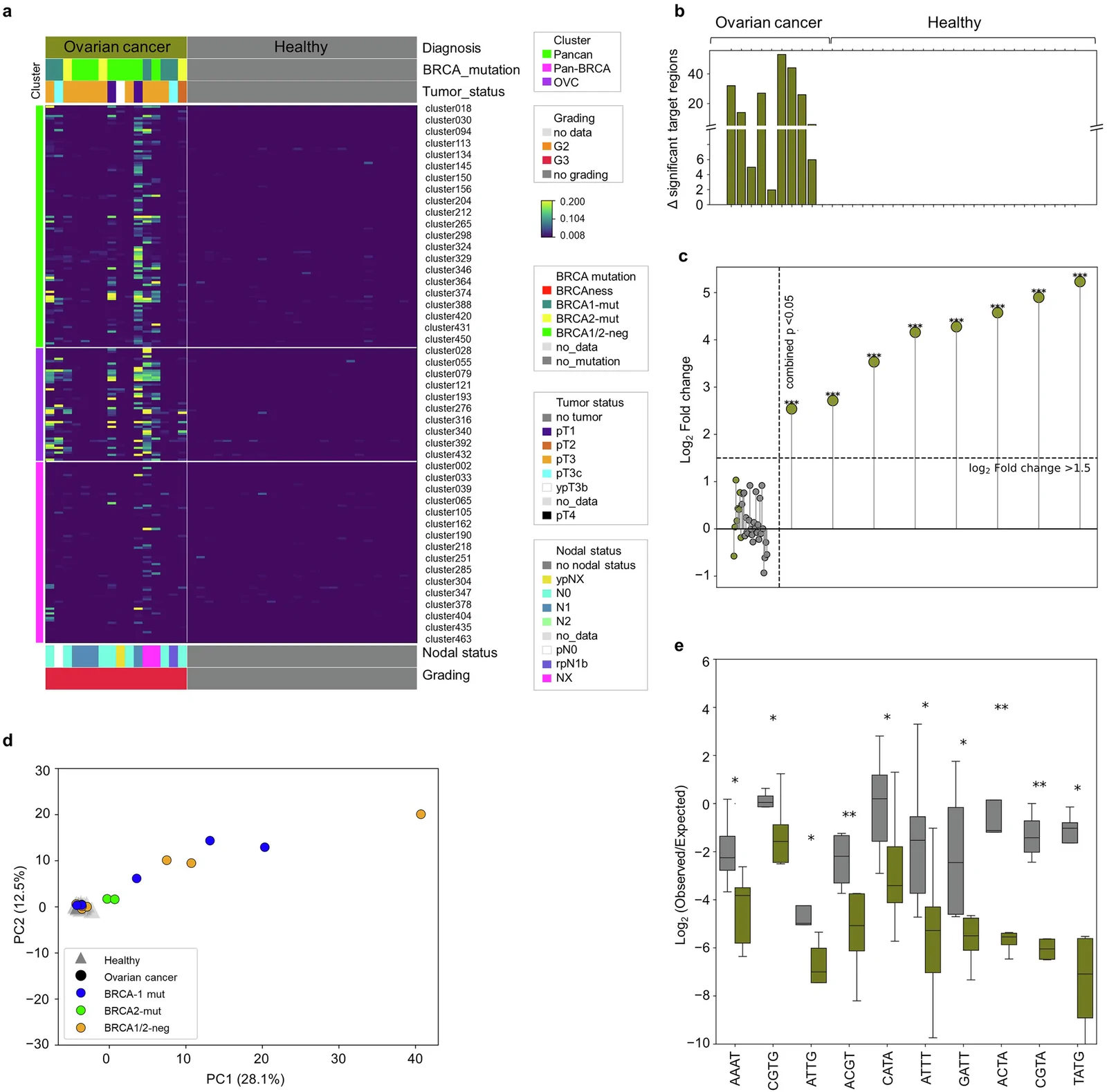
Hypermethylated ctDNA load in OC. **a** Clustermap showing differential hm-ctDNA fractions based on grading, nodal status and diagnosis in ovarian cancer vs healthy samples. **b** Barplot showing number of significant target regions identified in the OC samples compared to the healthy baseline. **c** Difference of hypermethylated fragments in per-sample effect size among the ovarian cancer and healthy control samples with established baseline. **d** Principal component analysis of the sample level clustering in OC. **e** Box plots showing the significant end-motifs identified with log2FC > 1.5 and *P*-value < 0.05.

OC samples exhibited pronounced enrichment of hm-ctDNA across multiple target regions. Heatmap visualization of hm-ctDNA load revealed clear separation between OC samples and healthy controls (Fig. 3a), with near-zero hm-ctDNA load observed in controls. At the regional level, OC-specific target regions showed higher hm-ctDNA load compared to BC and PanCan-derived regions, indicating tumor-type specificity. Notably, even early-stage tumors (stage I) exhibited hm-ctDNA fractions of up to 20% in a subset of regions.

At the sample level, 9 of 16 OC cases (62.5%) showed more than two significantly hypermethylated regions relative to controls (Fig. 3b). *BRCA1* positive samples tended to exhibit a higher number of significant regions (up to 44), whereas *BRCA2* positive samples showed weaker enrichment. However, significant hm-ctDNA signals were also observed in *BRCA1* and *BRCA2*-negative cases, indicating that fragment-level hypermethylation is not restricted to germline *BRCA1* and *BRCA2* carrier status.

Aggregated analysis across all regions identified 8 of 16 OC samples (50%) and zero healthy samples as significantly different from controls (*P* < 0.05, Log_2_FC > 1.5, see Fig. 3c), corresponding to a sensitivity of 50% (95% CI: 25.5–75.3%) at 100% specificity (95% CI: 86.8–100%). Principal component analysis (PCA) showed partial separation of OC samples from controls, but no distinct clustering by *BRCA1*/*2* carrier status (Fig. 3d).

End-motif analysis revealed minor differences between hm-ctDNA and norm-cfDNA in both OC (Fig. 3e). Among previously reported motifs, GGGA (OC) showed a mildly significant enrichment. When considered collectively, the predefined panel of 60 motifs (out of 256 possible 4mers) showed a strong depletion in OC (15.5% compared to 23.5% expectation), which, however mainly can be attributed to the significant or highly significant depletion of 10 motifs (CGTG, AAAT, ATTG, ACGT, CATA, ATTT, GATT, ACTA, CGTA and TATG).

### Fragment-level hypermethylation signals in breast cancer

**Fig. 4:**
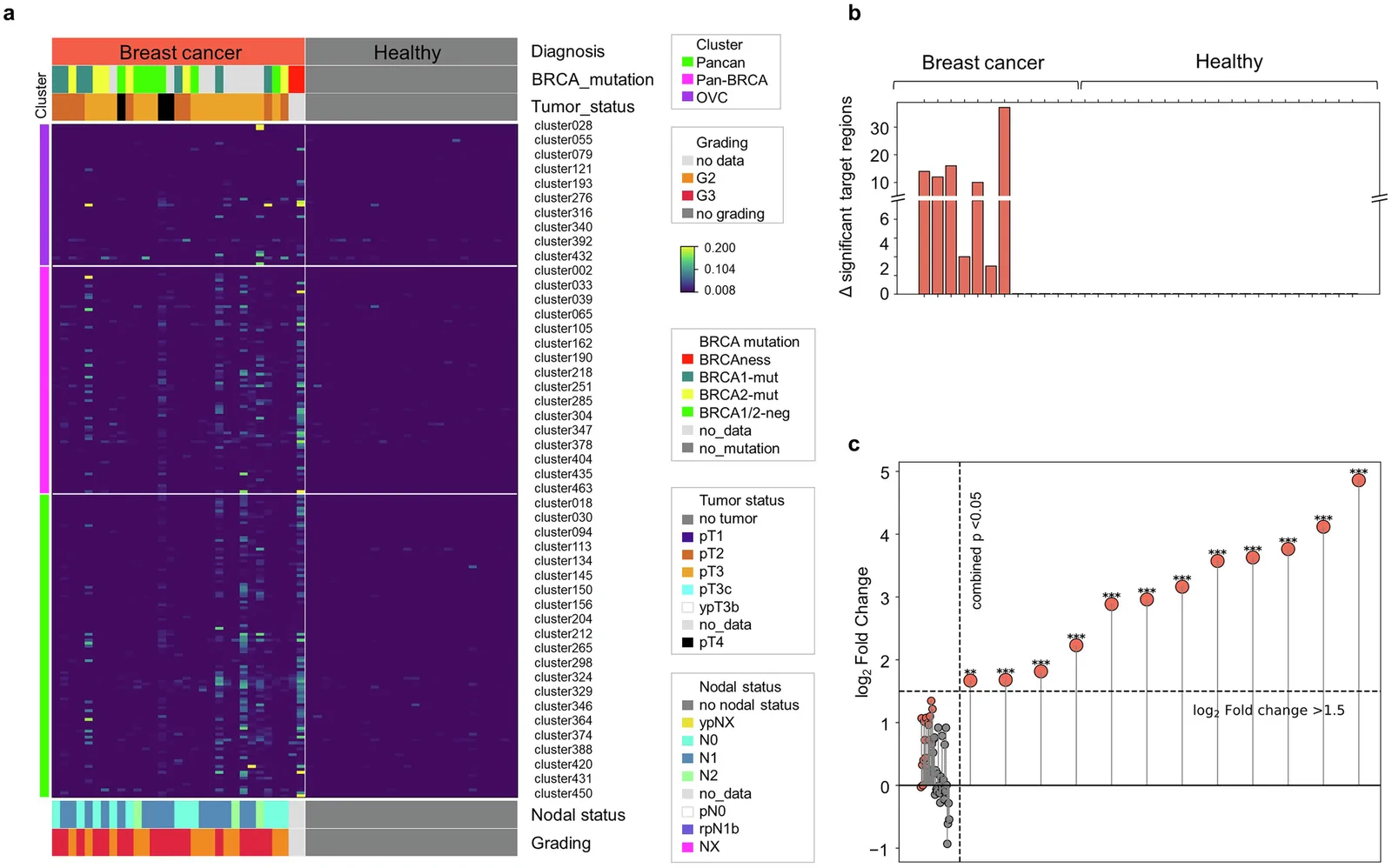
Hypermethylated ctDNA load in BC. **a** Clustermap showing differential hm-ctDNA fractions based on grading, nodal status and diagnosis in breast cancer vs healthy samples. **b** Barplot showing number of significant target regions identified in the BC samples compared to the healthy baseline. **c** Difference in hypermethylated fragments in per-sample effect size among the BC and healthy control samples with established baseline.

In BC, hm-ctDNA enrichment was observed in 12 of 31 samples (Fig. 4a), and generally at lower levels than in OC. Hm-ctDNA fractions per region ranged from 5% to 20% in enriched samples. At the target-region level, BC- and PanCan-specific regions showed stronger enrichment than OC-specific regions, consistent with tumor-type specificity. No significant enrichment was observed in healthy controls. At the sample level, several cases exhibited multiple significantly hypermethylated regions, including two *BRCA1* positive samples and one BRCAness case with particularly high signal (up to 37 regions; Fig. 4b).

Aggregated analysis of all regions identified 12 of 31 BC samples (38.7%) and no healthy samples as significantly different from controls (Fig. 4c), corresponding to a sensitivity of 38.7% (95% CI: 21.9–57.8%) at 100% specificity (95% CI: 86.8–100%). *BRCA1* positive carriers and BRCAness patients are the most frequent among the significant cases.

Interestingly, several cases show increased fold change but are not significant across all regions (Fig. 4c). This indicates that our panel is not optimal for BC and contains unspecific regions that introduce noise to the test. End-motif analysis in BC showed a mildly significant enrichment of the motif CCGC with no difference from the expected frequency in BC samples (24% vs 23.4% occurrence). This observation warrants further investigation in independent and larger cohorts.

Our results suggest that fragmentomic features such as length and end motifs might provide additional, albeit minor, discriminatory power compared to fragment-level hypermethylation. However, they have not been used to improve the detection rate in our test due to the inconsistency of the results and issues with using lack of motifs as evidence.

### Clinical and molecular factors influencing hm-ctDNA load

Across both cancer types, *BRCA1* positive samples showed a tendency toward higher hm-ctDNA burden compared to *BRCA2* positive and *BRCA1* and *BRCA2*-negative cases. In particular, *BRCA1* positive samples with nodal involvement (N1) exhibited increased numbers of hypermethylated regions, whereas N0 cases showed minimal enrichment (Fig. 4b). *BRCA2* positive samples generally displayed lower hm-ctDNA levels, with only one case showing moderate enrichment. A sample of a BRCAness tumor exhibited the highest hm-ctDNA burden (50 significant regions), suggesting that fragment-level hypermethylation reflects tumor biology beyond germline *BRCA1 / BRCA2* status.

Overall, these findings indicate that hm-ctDNA load is influenced by tumor biology, including *BRCA1* status, nodal involvement, and BRCAness, although variability within subgroups remains substantial.

## Discussion

Breast cancer (BC) and ovarian cancer (OC) remain major causes of cancer-related morbidity and mortality among women, particularly due to limitations in early detection strategies, most notably for OC^22,23^. This challenge is especially pronounced in individuals carrying pathogenic *BRCA1* or *BRCA2* variants, who face substantially increased heterogeneous cancer risk^24^. In carriers of pathogenic *BRCA1* and *BRCA2* variants, lifetime risks reach up to 72% for breast cancer and 44% (*BRCA1*) or 17% (*BRCA2*) for ovarian cancer, with risk modified by age, family history, and variant location^3^. While BC benefits from established screening programs, OC lacks effective population-wide early detection strategies. Genetic testing enables risk stratification, but liquid biopsies have emerged as a minimally invasive approach capable of capturing tumor-associated molecular alterations^25^. Importantly, beyond early detection, liquid biopsies also hold promise in advanced-stage disease for monitoring tumor burden, detecting recurrence, and characterizing metastatic heterogeneity, where tissue sampling is often impractical^26^.

Our study builds on previous work demonstrating the diagnostic potential of cfDNA methylation in BC and OC^13,27^, as well as published methods for detection of cancer-specific fragment-level hypermethylation^21^. However, our approach differs in several key aspects from previous methods. First, we opted for a targeted panel derived from existing tumor data instead of a low-coverage genome-wide approach. Second, the targeted approach allowed us to sequence ultra-deep (>10k coverage), enabling detection of even miniscule fractions of hypermethylated fragments. Importantly, incorporation of UMI-based error correction minimized sequencing artefacts, so sensitivity was driven primarily by recovery of unique molecules rather than sequencing error. Third, we focused on CpG-rich cfDNA fragments enabling robust detection of fragment-level hypermethylation. Finally, compared to previously published fragment-level hypermethylation approaches^21^, we employed a binary classification strategy (hypermethylated vs non-hypermethylated cfDNA fragments with high GC density) instead of machine learning methods, allowing for the use of conventional, robust, and easy to interpret statistical evaluation methods and for the analysis of small patient cohorts.

Though hypermethylation is often attributed to advanced stages of cancer, there are reports highlighting promoter hypermethylation and epigenetic silencing of tumor suppressor genes as important early events in breast cancer development^28,29^. We hypothesized that ultra-deep sequencing enables the detection of these characteristic changes even for low tumor burden at early cancer stages. Our results demonstrate that fragment-level hypermethylation is consistently enriched in cfDNA from patients with BC and OC and enables discrimination from healthy controls with high specificity (100%). Moreover, hypermethylated regions were largely tumor-type specific, showing enrichment of hm-ctDNA in regions that were selected based on hypermethylation in primary tumors of the same type.

While sensitivity remained moderate (38.7% for BC and 50% for OC), this performance is comparable to or exceeds several established liquid biopsy approaches. For example, CancerSEEK reported a sensitivity of 33% for BC^30^, while genome-wide fragmentation analysis using DELFI achieved 57% sensitivity at 98% specificity^31^. Similarly, the Galleri methylation-based test showed a sensitivity of 30.5% for BC at 99.5% specificity^32^. These comparisons suggest that the HBOC-specific panel designed in this study provides competitive performance for BC while maintaining perfect specificity in our cohort. Importantly, high specificity is valuable for establishing screening programs for high-risk populations such as *BRCA1* and *BRCA2* carriers, where the pre-test probability of disease is elevated. In this context, a highly specific assay could serve as a complementary “rule-in” test within surveillance programs, potentially improving early detection when combined with imaging-based screening.

We are not aware of published results concerning fragment-level hypermethylation cfDNA tests for ovarian cancer. Surprisingly, we observed stronger signals in OC than in BC, with 50% sensitivity on a samples level (test across all panel regions), and 62.5% when considering a minimum of two significant regions per sample. Presentation of enrichments per region via heat plots enables easy detection of OC cases simply by visual inspection (Fig. 3a). Moreover, the strong focus of enrichment in OC-specific regions facilitates the design of smaller and more specific panels for OC screening. These promising results therefore warrant replication studies in larger OC cohorts in the future.

The association between hm-ctDNA load and clinical features further supports the biological relevance of this approach. We observed a tendency toward higher hm-ctDNA burden in *BRCA1*-associated tumors, tumors with BRCAness signature, and in cases with nodal involvement, consistent with increased tumor burden and genomic instability. However, strong signals were also detected in some *BRCA1* and *BRCA2*-negative tumors, highlighting the broader applicability of this approach.

Beyond diagnostic performance, our findings provide insights into the biological characteristics of tumor-derived hypermethylated cfDNA. Hm-ctDNA fragments were enriched at ~165 bp and lacked the shorter fragment peak typically associated with hypomethylated regions. This is consistent with previous studies showing that hypomethylated regions preferentially generate shorter fragments due to increased nuclease accessibility^19^. Our results extend these observations by indicating that hypermethylated regions contribute to longer, nucleosome-protected cfDNA fragments, reinforcing the link between epigenetic state and cfDNA structure. We further explored end-motif signatures as an additional layer of cfDNA information and observed depletion of a subset of previously reported motifs in OC, which may be informative if validated in independent cohorts. However, in contrast to fragment-level hypermethylation, end-motif features showed limited discriminatory power. These findings suggest that fragmentomic- features derived from small, targeted panels provide complementary information but are insufficient as primary biomarkers. In contrast, genome-wide fragmentomics approaches have shown stronger performance^31^ and may enhance sensitivity when combined with methylation-based methods.

Several limitations should be considered. The cohort size is relatively small and enriched for advanced-stage tumors, which may limit generalizability and does not fully reflect early-stage scenarios. Especially for OC, only grade 3 tumors were available for testing. Therefore, sensitivity remains to be established in larger prospective cohorts for those cases, as well as the complementary value and clinical utility. Due to the limited size of our control cohort, we could not establish robust methylation ranges for healthy individuals. Hence, thresholds might need to be adjusted when the method is applied for screening of larger cohorts. In addition, our analysis focuses on hypermethylated regions, potentially overlooking informative hypomethylation signals. Moreover, a sizable fraction of the 360 selected target regions showed no signal, indicating that further refinement of the regions included in the panel could further improve the performance. Furthermore, although fragmentomic features showed enrichment in tumor-derived cfDNA, their standalone contribution to classification remained limited in this cohort and requires validation in larger datasets. Finally, we reported a 100% specificity with zero controls classified as cancer positive. However, given the limited number of healthy controls, the confidence interval is relatively wide (e.g., 95% CI: 86.8-100% for OC). Hence, our study cannot ascertain the specificity when applying our method for population-wide screening of thousands of individuals of risk.

Half of the studied patients did not show a significant characteristic methylation profile. The sensitivity could potentially be improved by integrating methylation and fragmentomics markers with ctDNA-based screening for characteristic oncogenic variants, e.g., for second hits in *BRCA1* or *BRCA2* of germline risk variant carriers. While this approach would significantly increase the panel size, extended multi-feature cfDNA analysis could compensate for the weaknesses of single biomarker strategies. Also, in this study we used panel-based EM-seq paired with ultra-deep NGS; but did not compare to the sensitivity of alternative methods. However, in-depth comparisons between enzymatic and bisulfite conversion combined with NGS or ddPCR approaches have been performed previously^33–35^.

Despite these limitations, our study provides strong evidence that fragment-level hypermethylation represents a biologically meaningful biomarker for ctDNA detection in HBOC. The targeted design reduces sequencing requirements compared to genome-wide approaches and enables ultra-deep sequencing, making this strategy potentially more sensitive, scalable, and cost-effective. Future studies should evaluate this approach in longitudinal and prospective screening settings, particularly in high-risk populations such as *BRCA1* and *BRCA2* mutation carriers.

In summary, we present a targeted approach for ultra-deep sequencing and detection of hypermethylated CpG-rich cfDNA fragments, enabling the accurate detection of tumor-derived cfDNA in patients with breast and ovarian cancer. By interrogating CpG methylation proportions in CpG-rich cfDNA fragments with ultra-deep NGS, this method captures coordinated epigenetic patterns that are not accessible through conventional CpG-level analyses. While sensitivity remains moderate, the high specificity and clinical interpretability of this approach highlight its potential as a complementary tool for non-invasive cancer detection, particularly in high-risk populations. These findings establish hypermethylation on tumor-derived CpG-rich cfDNA in plasma as a promising new dimension in liquid biopsy analysis and provide a foundation for future clinical validation studies.

## Methods

### Patient recruitment and sample collection

Blood samples from healthy individuals were obtained from a commercial biobank (researchdonors.co.uk), while samples from breast cancer (BC) and ovarian cancer (OC) patients were collected from the in-house biobank of the Department of Gynecology at the University Hospital Tübingen, prioritizing carriers of pathogenic *BRCA1* and *BRCA2* germline variants (Supplementary Fig. 1a). BRCAness status was extracted from health records following external routine testing. All samples were collected in EDTA tubes. Informed consent was obtained from all the participants. The study was conducted under the LIQUID biopsy METhylomics to advance Cancer precision Medicine (Liquid Metcam) under the ethics approval number 409/2022BO2.

Plasma was isolated using a two-step centrifugation protocol (2000 × *g* for 10 min) and stored at −80 °C. Cell-free DNA (cfDNA) was extracted from 1 to 2 mL of plasma using the MagMAX cfDNA Isolation Kit (Thermo Fisher, cat. #A29319) according to the manufacturer’s instructions. cfDNA was eluted in 54 µL of elution buffer and quantified using the Qubit dsDNA High Sensitivity Assay (Invitrogen, cat. #Q32851). Fragment size distribution and quality were assessed using the Agilent high sensitivity cell-free DNA ScreenTape assay D1000 (cat.# 5067-5630) on the Agilent 4200 TapeStation system.

### Design of a targeted CpG panel for HBOC

To identify cancer-specific hypermethylated regions, we analyzed 375,094 CpG sites from publicly available 450k methylation array datasets, including the TCGA breast cancer (*BRCA*) cohort, a high-grade serous ovarian cancer (GSE72021), and Fallopian tube fimbriae tissue datasets from *BRCA1* and *BRCA2* mutation carriers (GSE74845) for ovarian cancer. Additionally, cfDNA methylation profiles from healthy individuals (GSE122126) were used to identify background signals^36^.

CpG sites were filtered to identify regions that are (i) consistently unmethylated in normal tissues and (ii) hypermethylated in BC, OC, or across multiple cancer types (PanCan). Filtering criteria included:

- removal of CpGs with missing values
- minimal difference between cancer and normal tissues: min(cancer) − max(normal) ≥ 0.1
- low background methylation in normal tissues: max(normal) < 0.2
- exclusion of CpGs with >10% methylation in healthy cfDNA

Filtered CpGs were clustered into regions with a maximum inter-CpG distance of 180 bp, including all adjacent CpG, reflecting the size of cfDNA fragments. From each category (BC, OC, PanCan), 120 regions were selected, resulting in a total panel of 360 regions. The regions are selected such that they are enriched in promoters, gene bodies, and transcription start sites, which are known to undergo early tumor-associated methylation changes. For BC we prioritized characteristic regions hypermethylated in both basal-like and luminal subtypes frequently associated with *BRCA1* and *BRCA2* associated breast cancers, respectively^37^. Additionally, 24 SNPs (non-C positions; allele frequency of 0.30–0.70 in population) were included for sample identity verification and quality control (see Supplementary Table 1). Characteristics of selected CpGs and regions are shown in Supplementary Table 2 and Supplementary Fig. 3.

### Enzymatic methylation sequencing

DNA methylation library preparation was initiated using 20 ng of cfDNA input using the NEBNext Enzymatic Methyl-seq Kit (New England Biolabs, cat. #E7120)^38^. After end repair and dA-tailing, methylated unique molecular identifier (UMI) adapters (Twist Bioscience, cat. #105040) were ligated to DNA fragments. Adapter cytosines were pre-methylated to prevent enzymatic conversion and enable accurate deduplication. Target enrichment was performed using the custom-designed panel, described previously, and the Twist Fast Hybridization Kit (Twist Bioscience, cat. #100938). Libraries were amplified and sequenced in paired-end mode (2 × 100 bp) on Illumina NovaSeq 6000 and NovaSeq X Plus platforms^39^ to a depth of > 10.000x coverage.

### Bioinformatic processing

The complete workflow for sample data processing and analysis is shown in Supplementary Fig. 1. In brief, sequencing reads were quality filtered and adapter-trimmed using SeqPurge (v2024_08-11)^40^. UMI sequences were extracted using fgbio, and reads were aligned to the human reference genome (GRCh38) using Bowtie2 (v2.5.3).

Aligned reads were grouped by UMI and processed with fgbio (v3.1.1) ^41^to generate duplex consensus reads. BAM files were sorted and indexed using samtools (v1.23). Methylation calling was performed using the Bismark methylation extractor (v0.25.1)^42^. Overlapping read pairs were clipped using BamClipOverlap, and per-read methylation metrics were computed using MethylDackel (v0.4.0) (https://github.com/dpryan79/methyldackel). Reads showing incomplete conversion were removed to minimize false-positive methylation signals.

### Identification of fragment-level hypermethylated ctDNA

To detect tumor-derived cfDNA, we developed a fragment-level methylation analysis strategy that provides robust and easy to interpret results for clinical applications. Only cfDNA fragments with lengths between 125 bp and 180 bp were retained, corresponding to mono-nucleosomal fragments. Fragments were further filtered to include only those containing at least six CpG sites, enabling robust estimation of methylation density per fragment. A fragment was classified as hypermethylated ctDNA (hm-ctDNA) if ≥75% of its CpG sites were methylated. Fragments not meeting this criterion (i.e., fragments with ≥ 6 CpG but < 75% methylated CpG) were classified as normal cfDNA (norm-cfDNA). This binary classification (as explained in Fig. 1b) of fragment-level hypermethylation enables the application of robust statistical measures such as fraction (ctDNA load), fold-change, and significance of hm-ctDNA enrichment in a single region or across all regions when comparing affected to healthy patient groups. For each target region, hm-ctDNA load was defined as the fraction of hm-ctDNA fragments among all eligible cfDNA fragments within that region:

$${hm}{{{\rm{\_}}}}{ctDNA}\,{load}=\sum {hm}{{{\rm{\_}}}}{ctDNA}/\left(\sum {hm}{{{\rm{\_}}}}{ctDNA}+\sum {norm}{{{\rm{\_}}}}{cfDNA}\right)$$

### Fragment length and end-motif analysis

To explore structural features of hm-ctDNA fragments, we analyzed their length distribution (using histograms) and sequence motifs at fragment ends. For the latter, the first four nucleotides at the 5′ end of each fragment were extracted, resulting in 256 possible motifs^43^. Motif enrichment was calculated separately for hm-ctDNA and norm-cfDNA fragments using observed-to-expected ratios relative to the GRCh38 reference genome. Within-sample comparisons were used to minimize GC bias^44^.

We evaluated a predefined panel of 60 motifs previously reported in BC and OC^45–47^. Motifs with log2(observed/expected) > 1.2 were considered enriched, while motifs with log2(observed/expected) < 0.8 were considered depleted.

### Statistical analysis

Batch effects were corrected using pyComBat (v3.12.1). A healthy control cohort (*n* = 26) was used to establish baseline methylation levels for each target region. For normalization of all samples, we applied the mean and standard deviation of beta values obtained for healthy samples^15^.

To assess region-level enrichment of hm-ctDNA, 2 × 2 contingency tables comparing hm-ctDNA and norm-cfDNA fragments were constructed relative to the healthy cohort. To compute enrichment for healthy samples we opted for a leave-one-out strategy, comparing the focal sample to the remaining healthy cohort. Statistical significance was evaluated using Fisher’s exact test, and combined *P*-values were calculated per sample across all regions.

Group-level differences between healthy controls, BC, and OC samples were assessed using the Mann–Whitney *U* test^48^. *P*-values were adjusted using the Benjamini–Hochberg method. Significance in figures is indicated with stars ‘****’ for *P*-values < 0.0001, ‘***’ for *P*-values < 0.001, ‘**’ for *P*-values < 0.01 and ‘*’ for *P*-values < 0.05). Non-significant values are removed from the figures. Hm-ctDNA load and motif enrichment were visualized using pyComplexHeatmap (v1.8.3), and principal component analysis (PCA) was performed using scikit-learn (v1.8.0). Sensitivity and specificity were calculated with exact 95% confidence intervals using the Clopper-Pearson method.

## Supplementary information

Sekar_2026_npj_Breast_Cancer_Supplement_revised.

## Data Availability

The datasets generated and/or analyzed during the current study are not publicly available due to requirements for the protection of patient information. However, the datasets are available from the corresponding author upon reasonable request, subject to applicable privacy and ethical requirements.

## Code availability

All the code and scripts used in the study were written in Python v3.12.2 and processed with Jupyter Notebook v3.2.1. They are available from the GitHub link https://github.com/imgag/hm-ctDNA.
