# Supplementary material for: Hypermethylation in CpG-rich cfDNA fragments enables accurate detection of circulating tumor DNA in BRCA1- and BRCA2-associated breast and ovarian cancer
Source: NPJ Breast Cancer. 2026 Nov 11;12:145. doi: 10.1038/s41523-026-01076-y (PMC13754801; doi:10.1038/s41523-026-01076-y)
Supplement: Supplementary file 1 [file 41523_2026_1076_MOESM1_ESM.pdf]

Supplementary figures and tables

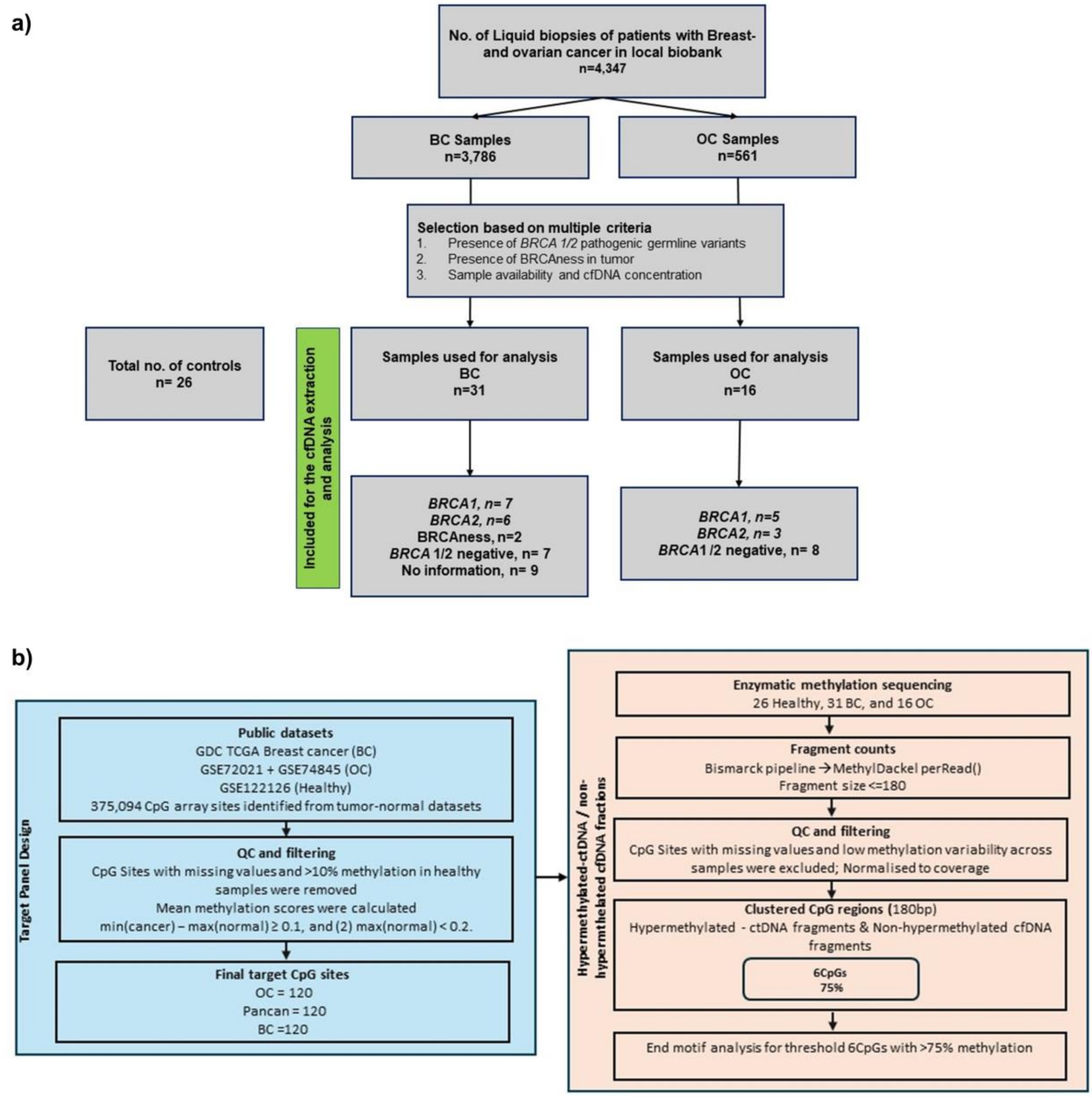

Supplementary Figure 1. a) consort diagram of patient included in the study. b)Flowchart of the targeted NGS method for detection of hypermethylation in CpG-rich cfDNA fragments. All processing steps from target panel design and the bioinformatics processing.

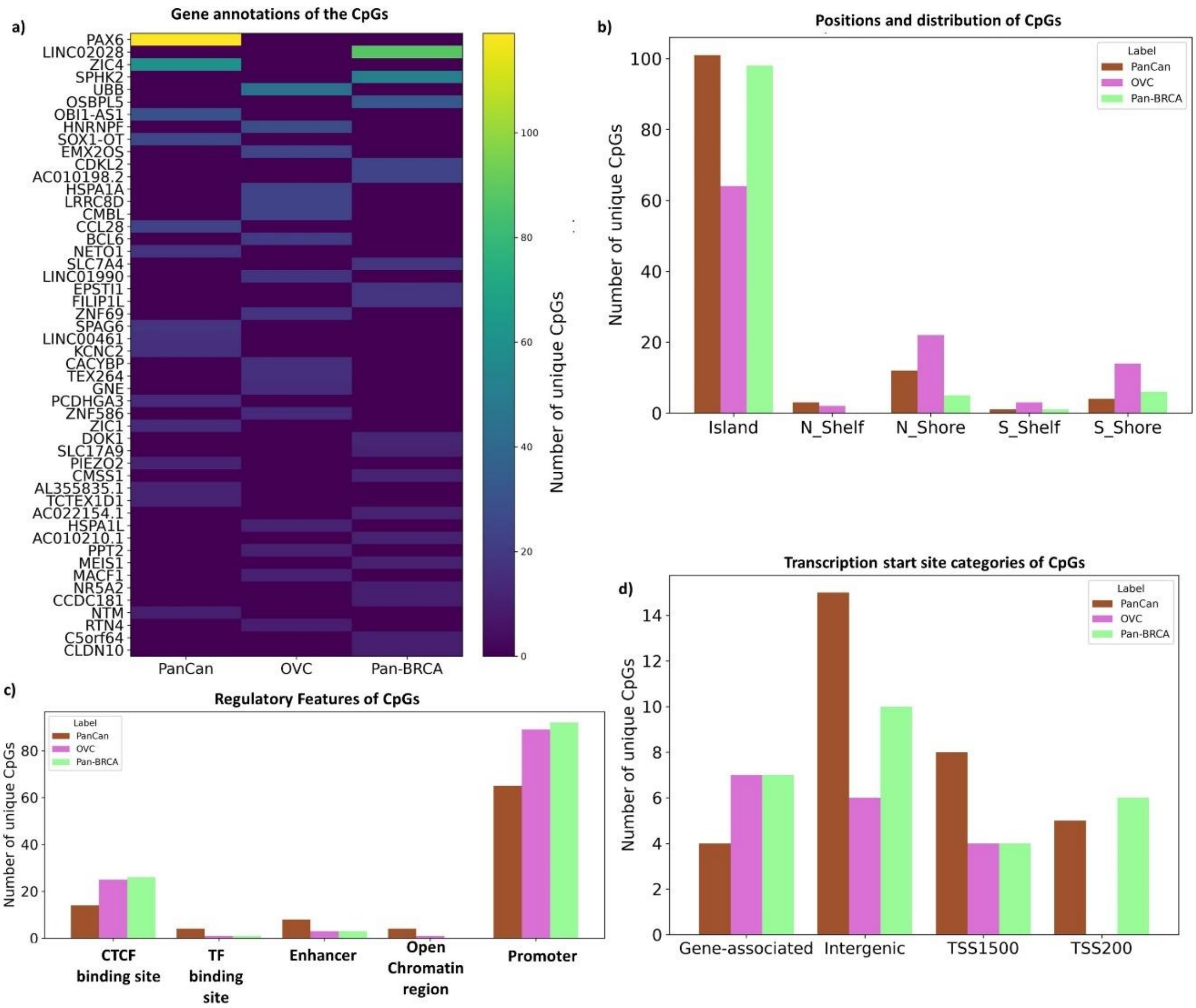

Supplementary Figure 2. Genomic annotation of the target region. a) Genes or gene-promoters overlapping with the target regions in OC, BC or PanCan. b) Number of targeted CpGs overlapping CpG islands, shores or shelves. c) Number of targeted CpGs overlapping promoters, open chromatin region, enhancer, transcription factor binding and CTCF binding sites. d) Number of targeted CpGs that are associated to genes, intergenic regions, TSS 1500 and TSS 200 regions.

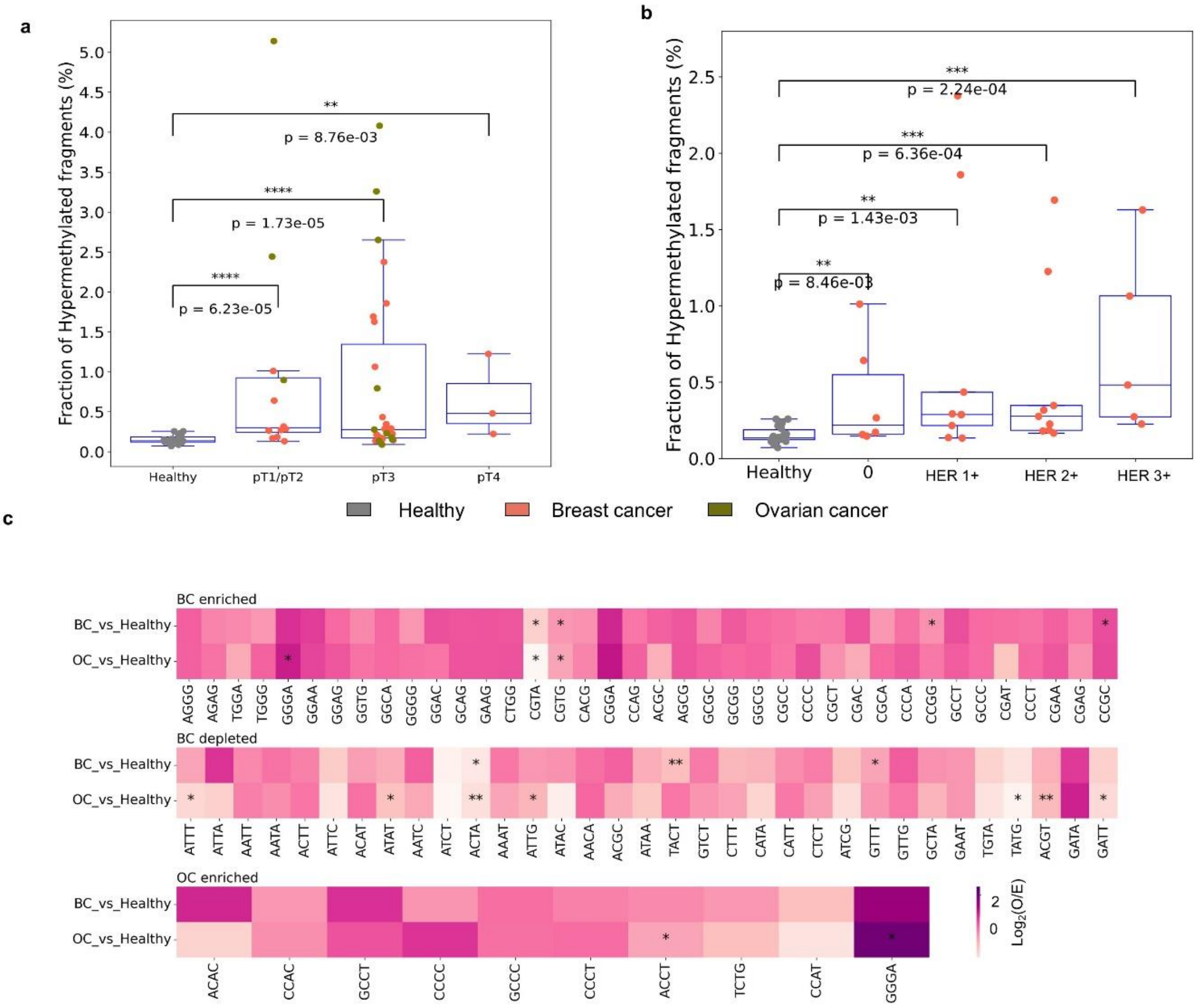

Supplementary Figure 3: (a) Fraction of hypermethylated-ctDNA fragments with respect to tumor stage. (b) Fraction of hypermethylated-ctDNA fragments with respect to HER2 positive status (c)Heatmap of significant End-motif identified in the cohort in comparison to the established end motifs computed based on the observed to expected ratio (O/E) from breast cancer vs healthy and ovarian cancer vs healthy;

Supplementary Table S1. Characteristics of the fingerprint SNPs included in the panel and used in sample identification verification and quality control, chr = chromosome

No.	chr	start	end	SNP and Allele frequency
**1**	chr1	158677731	158677732	SNP_A/G_AF=0.55004
**2**	chr11	534241	534242	SNP_A/G_AF=0.31570
**3**	chr12	48654954	48654955	SNP_G/T_AF=0.58123
**4**	chr16	58511521	58511522	SNP_A/G_AF=0.65908
**5**	chr17	41571563	41571564	SNP_T/G_AF=0.63357
**6**	chr17	41567737	41567738	SNP_T/A_AF=0.63407
**7**	chr19	39885882	39885883	SNP_G/A_AF=0.33360
**8**	chr19	2226676	2226677	SNP_G/A_AF=0.35035
**9**	chr19	3110350	3110351	SNP_T/G_AF=0.41704
**10**	chr19	4101063	4101064	SNP_G/T_AF=0.47828
**11**	chr3	52609290	52609291	SNP_A/G_AF=0.43530
**12**	chr5	38958647	38958648	SNP_A/G_AF=0.40168
**13**	chr5	35875490	35875491	SNP_A/T_AF=0.48847
**14**	chr5	14290695	14290696	SNP_G/A_AF=0.50354
**15**	chr5	35871087	35871088	SNP_G/A_AF=0.63977
**16**	chr5	31401339	31401340	SNP_A/G_AF=0.68752
**17**	chr6	30925650	30925651	SNP_G/A_AF=0.31781
**18**	chr6	29975205	29975206	SNP_G/A_AF=0.53267
**19**	chr8	11850845	11850846	SNP_G/A_AF=0.35793
**20**	chr9	32425911	32425912	SNP_A/G_AF=0.34503
**21**	chr9	35674055	35674056	SNP_G/A_AF=0.37372
**22**	chr9	21816758	21816759	SNP_G/A_AF=0.39617
**23**	chr9	133639991	133639992	SNP_A/G_AF=0.44983
**24**	chr9	5081779	5081780	SNP_G/A_AF=0.52651

Supplementary Table 2. Annotation of the 360 CpG biomarkers identified as hypermethylated for BC or OC in the TCGA cohort. These CpGs were clustered into target regions with a maximum inter-CpG distance of 180 bp, including all adjacent CpGs. Annotations include genomic locus, cancer type and subtype, as well as overlap to TSS, CpG islands and regulatory features based on the Ensembl database.

No	CpG	chr	region- start	region- end	label	Gene	TSS Category	CpG Island Position	Regulatory Feature
1	cg07113230	chr1	3068747	3068749	Pan-BRCA		Intergenic	Island	promoter
2	cg22393461	chr1	8217333	8217335	OVC	NMNAT1	Unknown		CTCF binding site
3	cg09299086	chr1	9950599	9950601	OVC	RCC2	Unknown	Island	CTCF binding site
4	cg00478361	chr1	17419941	17419943	Pan-BRCA	ZPLD2P	Unknown	Island	promoter
5	cg05307957	chr1	26225624	26225626	OVC	ARID1A	Unknown		
6	cg13863668	chr1	26702482	26702484	OVC	GMEB1	Unknown	S_Shore	promoter
7	cg19856520	chr1	28670048	28670050	OVC	AL442071.2	Unknown		
8	cg02241397	chr1	39231003	39231005	Pan-BRCA	TAL1	Unknown	Island	promoter
9	cg04501188	chr1	47232408	47232410	OVC	FOXD2	TSS1500	Island	promoter
10	cg12640000	chr1	47438498	47438500	PanCan	L1TD1	TSS1500	Island	promoter
11	cg15617155	chr1	62195068	62195070	PanCan		Intergenic	Island	promoter
12	cg06962177	chr1	63319649	63319651	PanCan		Intergenic	Island	promoter
13	cg10249375	chr1	63320274	63320276	PanCan		Intergenic	Island	enhancer
14	cg09318375	chr1	63330262	63330264	PanCan	TCTEX1D1	Unknown	Island	promoter
15	cg25339566	chr1	66752406	66752593	PanCan	TCTEX1D1	Unknown	Island	promoter
16	cg24161501	chr1	66752406	66752593	PanCan	TCTEX1D1	Unknown	Island	promoter
17	cg15825692	chr1	66752406	66752593	Pan-BRCA	DNAI3	Unknown		promoter
18	cg22488158	chr1	85062259	85062362	Pan-BRCA	DNAI3	Unknown		promoter
19	cg24353213	chr1	85062259	85062362	OVC	AC093423.3	Unknown	N_Shore	promoter
20	cg14565265	chr1	89843060	89843062	OVC	AC093423.3	Unknown	Island	promoter
21	cg10463708	chr1	89843391	89843393	OVC	AC093423.3	Unknown	Island	promoter
22	cg04436714	chr1	89843645	89843660	OVC	AC093423.3	Unknown	Island	promoter
23	cg24884703	chr1	89843645	89843660	PanCan	AC092805.1	Unknown	Island	
24	cg26436330	chr1	90719864	90719866	Pan-BRCA	COL11A1	Unknown		promoter
25	cg01016662	chr1	103108143	103108145	Pan-BRCA		Intergenic	Island	CTCF binding site
26	cg09819083	chr1	110130459	110130558	Pan-BRCA		Intergenic	Island	CTCF binding site
27	cg00977827	chr1	110130459	110130558	Pan-BRCA		Intergenic		CTCF binding site
28	cg03539016	chr1	143698092	143698197	Pan-BRCA		Intergenic		CTCF binding site
29	cg18943693	chr1	143698092	143698197	Pan-BRCA	AL691442.1	Gene-associated	Island	CTCF binding site
30	cg26248173	chr1	155071084	155071086	Pan-BRCA		Intergenic	Island	CTCF binding site
31	cg00100121	chr1	166884340	166884342	Pan-BRCA	CCDC181	Unknown	Island	promoter
32	cg00002719	chr1	169427396	169427469	Pan-BRCA	CCDC181	Unknown	Island	promoter
33	cg13673319	chr1	169427396	169427469	OVC	CACYBP	Unknown	N_Shore	promoter
34	cg13384453	chr1	174999196	174999252	OVC	CACYBP	Unknown	N_Shore	promoter
35	cg02275294	chr1	174999196	174999252	OVC	SOAT1	Unknown	N_Shore	promoter
36	cg06515144	chr1	179293326	179293328	Pan-BRCA	NPHS2	Unknown	Island	
37	cg13931925	chr1	179575955	179576082	Pan-BRCA	NPHS2	Unknown	Island	
38	cg05391318	chr1	179575955	179576082	Pan-BRCA	NR5A2	Unknown	Island	promoter
39	cg00001583	chr1	200042555	200042659	Pan-BRCA	NR5A2	Unknown	Island	promoter
40	cg14221039	chr1	200042555	200042659	OVC	LEMD1	Unknown	S_Shore	CTCF binding site
41	cg00761787	chr1	205430991	205430993	OVC	AC244035.1	Unknown	Island	CTCF binding site

42	cg20242778	chr1	206117628	206117630	OVC	AL445423.3	Unknown		CTCF binding site
43	cg21710324	chr1	220810317	220810319	OVC	TMEM63A	TSS1500	N_Shore	promoter
44	cg19306380	chr1	225881488	225881490	OVC	AL353593.1	Unknown	Island	promoter
45	cg13409449	chr10	228275558	228275560	OVC	GATA3	Unknown	Island	promoter
46	cg05099508	chr10	8055390	8055392	PanCan	SPAG6	Unknown	Island	
47	cg24031355	chr10	22345502	22345650	PanCan	SPAG6	Unknown	Island	
48	cg10648197	chr10	22345502	22345650	PanCan	SPAG6	Unknown	Island	
49	cg14776321	chr10	22345502	22345650	Pan-BRCA	FAM238C	Unknown		
50	cg07638650	chr10	26931511	26931513	Pan-BRCA	RASGEF1A	Unknown	Island	CTCF binding site
51	cg10765471	chr10	43202400	43202402	Pan-BRCA	LINC02633	TSS1500	Island	promoter
52	cg04424930	chr10	43322980	43322982	OVC	HNRNPF	Unknown	Island	promoter
53	cg17824240	chr10	43397341	43397568	OVC	HNRNPF	Unknown	Island	promoter
54	cg09936190	chr10	43397341	43397568	OVC	HNRNPF	Unknown	S_Shore	promoter
55	cg05525499	chr10	43397341	43397568	OVC	HNRNPF	Unknown	S_Shore	promoter
56	cg01701415	chr10	43397341	43397568	OVC	NUDT13	Unknown		promoter
57	cg16178415	chr10	73110552	73110554	OVC	TSPAN14	Unknown		CTCF binding site
58	cg26596964	chr10	80505688	80505690	Pan-BRCA	PAX2	TSS200	Island	
59	cg17138769	chr10	100735596	100735598	PanCan	VAX1	Gene-associated	Island	
60	cg26288991	chr10	117132699	117132701	OVC	EMX2OS	Unknown	N_Shore	open_chromatin_region
61	cg09176256	chr10	117533695	117533697	OVC	EMX2OS	Unknown	Island	CTCF binding site
62	cg16063747	chr10	117534455	117534457	OVC	EMX2OS	Unknown	Island	CTCF binding site
63	cg11854504	chr10	117534661	117534663	OVC	EMX2OS	Unknown	Island	
64	cg23932491	chr10	117535208	117535210	OVC	EMX2OS	Unknown	Island	
65	cg05627029	chr10	117535544	117535546	Pan-BRCA	AC010998.1	Gene-associated	Island	CTCF binding site
66	cg11171221	chr10	120949018	120949020	PanCan		Intergenic	S_Shore	
67	cg03109827	chr10	123143327	123143329	PanCan	TCERG1L	TSS1500	Island	CTCF binding site
68	cg20090551	chr11	131312085	131312087	OVC	TSSC4	Unknown	Island	promoter
69	cg22074576	chr11	2400060	2400062	Pan-BRCA	OSBPL5	Unknown	Island	promoter
70	cg11678461	chr11	3160495	3160699	Pan-BRCA	OSBPL5	Unknown	Island	promoter
71	cg23357130	chr11	3160495	3160699	Pan-BRCA	OSBPL5	Unknown	Island	promoter
72	cg16865446	chr11	3160495	3160699	PanCan	PAX6	Unknown	S_Shore	
73	cg22557091	chr11	31800753	31800755	PanCan	PAX6	Unknown	Island	
74	cg09182533	chr11	31806371	31806373	Pan-BRCA	MACROD1	Unknown	N_Shore	CTCF binding site
75	cg26840755	chr11	64007598	64007600	OVC	NEAT1	Gene-associated		promoter
76	cg09703323	chr11	65430304	65430406	OVC	NEAT1	Gene-associated		promoter
77	cg15617814	chr11	65430304	65430406	PanCan	NTM	Unknown	Island	promoter
78	cg12064372	chr12	131910597	131910599	Pan-BRCA	AC010198.2	Unknown	Island	promoter
79	cg03476860	chr12	30795857	30795861	Pan-BRCA	AC010198.2	Unknown	Island	promoter
80	cg25810484	chr12	30795857	30795861	Pan-BRCA		Intergenic	S_Shore	CTCF binding site
81	cg11560280	chr12	53695701	53695714	Pan-BRCA		Intergenic	S_Shore	CTCF binding site
82	cg08803500	chr12	53695701	53695714	Pan-BRCA	HOXC13	Gene-associated	Island	CTCF binding site
83	cg21707187	chr12	53945039	53945041	PanCan		Intergenic	Island	
84	cg03078269	chr12	53952077	53952079	PanCan		Intergenic	Island	open_chromatin_region
85	cg00660843	chr12	53961189	53961191	Pan-BRCA	PDE1B	Unknown		promoter
86	cg00986824	chr12	54560615	54560617	PanCan	KCNC2	Unknown	Island	TF binding site
87	cg17203063	chr12	75207684	75207686	PanCan	KCNC2	Unknown	Island	
88	cg23535137	chr12	75207930	75207932	OVC	CRADD	Unknown		enhancer
89	cg21851351	chr13	93784675	93784677	PanCan	GSX1	TSS1500	Island	

90	cg19587904	chr13	27793949	27793951	Pan-BRCA	PLUT	TSS1500	Island	
91	cg03344149	chr13	27918287	27918289	OVC	CDX2	Gene-associated	Island	promoter
92	cg22125968	chr13	27961421	27961423	Pan-BRCA	EPSTI1	Unknown	Island	promoter
93	cg01536987	chr13	42992335	42992357	Pan-BRCA	EPSTI1	Unknown	Island	promoter
94	cg06679720	chr13	42992335	42992357	PanCan	DLEU1	Unknown	Island	promoter
95	cg26132774	chr13	50843549	50843551	PanCan	AL445209.1	Unknown	Island	promoter
96	cg24221648	chr13	78596010	78596149	PanCan	AL445209.1	Unknown	Island	promoter
97	cg19497031	chr13	78596010	78596149	PanCan	OBI1-AS1	Unknown		promoter
98	cg16232183	chr13	78602136	78602138	Pan-BRCA	CLDN10	Unknown	Island	CTCF binding site
99	cg06428163	chr13	95552599	95552620	Pan-BRCA	CLDN10	Unknown	Island	CTCF binding site
100	cg04603730	chr13	95552599	95552620	Pan-BRCA	CLDN10	Unknown	Island	CTCF binding site
101	cg10305311	chr13	95552599	95552620	Pan-BRCA	CLDN10	Unknown	Island	CTCF binding site
102	cg16556145	chr13	95552599	95552620	Pan-BRCA	CLDN10	Unknown	Island	promoter
103	cg00529958	chr13	95552929	95552931	PanCan	ZIC5	TSS200	Island	promoter
104	cg00295794	chr13	99972033	99972035	PanCan		Intergenic	Island	
105	cg08152546	chr13	99989154	99989156	PanCan	SOX1-OT	Unknown	Island	CTCF binding site
106	cg20829347	chr13	112062892	112062894	PanCan	SOX1	Unknown	Island	promoter
107	cg00663972	chr13	112066612	112066614	PanCan	SOX1	Unknown	Island	promoter
108	cg15466862	chr13	112067410	112067412	PanCan	SOX1	Unknown	Island	promoter
109	cg04047221	chr13	112068018	112068020	PanCan	SOX1	Unknown	Island	promoter
110	cg15415452	chr13	112069162	112069164	PanCan	SOX1-OT	Unknown	Island	promoter
111	cg21054521	chr13	112105040	112105073	PanCan	SOX1-OT	Unknown	Island	promoter
112	cg13692446	chr13	112105040	112105073	PanCan	SOX1-OT	Unknown	Island	promoter
113	cg01566235	chr14	112105404	112105406	PanCan	AL132857.1	Unknown	Island	CTCF binding site
114	cg05057720	chr14	36521895	36521897	PanCan	AL355835.1	Unknown	Island	CTCF binding site
115	cg23288059	chr14	38255469	38255471	PanCan	AL355835.1	Unknown	Island	CTCF binding site
116	cg18479593	chr14	38255739	38255741	PanCan		Intergenic	Island	
117	cg02036261	chr14	56798841	56798843	PanCan	OTX2	Unknown	Island	promoter
118	cg21039708	chr14	56809338	56809340	PanCan	OTX2	Unknown	Island	enhancer
119	cg12189551	chr14	56812010	56812012	PanCan	C14orf39	Unknown	Island	promoter
120	cg13539545	chr14	60486226	60486228	PanCan	C14orf39	Gene-associated	Island	open_chromatin_region
121	cg14876708	chr14	60507104	60507106	OVC	PLEKHH1	Unknown	N_Shore	CTCF binding site
122	cg01693026	chr14	67571403	67571405	OVC	DIO3	Unknown	Island	promoter
123	cg23071808	chr14	101560615	101560617	Pan-BRCA	LINC02323	Gene-associated	Island	
124	cg22796749	chr14	102555304	102555306	OVC		Intergenic	Island	CTCF binding site
125	cg18985581	chr14	105045733	105045970	OVC		Intergenic	Island	CTCF binding site
126	cg09197895	chr14	105045733	105045970	OVC		Intergenic	Island	CTCF binding site
127	cg08727193	chr14	105045733	105045970	OVC		Intergenic	Island	CTCF binding site
128	cg17911021	chr15	105045733	105045970	OVC	AC051619.7	Unknown	S_Shelf	promoter
129	cg25587091	chr15	45201217	45201219	OVC	RAB11A	Unknown		promoter
130	cg11601252	chr15	65833124	65833126	PanCan	SKOR1	Unknown	Island	
131	cg21523564	chr15	67829800	67829802	Pan-BRCA	SCAMP5	Gene-associated	Island	promoter
132	cg06702537	chr16	74959149	74959151	OVC	USP7	Unknown	N_Shelf	promoter
133	cg05581701	chr16	8960053	8960055	Pan-BRCA	MT1M	Unknown	Island	promoter
134	cg00916884	chr16	56632410	56632423	Pan-BRCA	MT1M	Unknown	Island	promoter
135	cg18438944	chr16	56632410	56632423	Pan-BRCA	MT1A	TSS200	S_Shore	promoter
136	cg26970841	chr16	56638612	56638614	Pan-BRCA	IRF8	Unknown	Island	promoter
137	cg24720583	chr17	85899059	85899061	Pan-BRCA	BCL6B	Unknown	Island	CTCF binding site

138	cg06700146	chr17	7022874	7022876	Pan-BRCA	BCL6B	Unknown	Island	CTCF binding site
139	cg02346492	chr17	7023080	7023082	OVC	NTN1	Gene-associated	Island	promoter
140	cg08547691	chr17	9239856	9239858	OVC	NTN1	Gene-associated	Island	promoter
141	cg14349843	chr17	9240436	9240438	OVC	UBB	Unknown	Island	promoter
142	cg05090851	chr17	16380783	16381195	OVC	UBB	Unknown	Island	promoter
143	cg24668364	chr17	16380783	16381195	OVC	UBB	Unknown	Island	promoter
144	cg25745651	chr17	16380783	16381195	OVC	UBB	Unknown	Island	promoter
145	cg03954587	chr17	16380783	16381195	OVC	UBB	Unknown	Island	promoter
146	cg15761609	chr17	16380783	16381195	OVC	FAM20A	Unknown	S_Shore	
147	cg03602280	chr18	68601925	68601927	PanCan	PIEZO2	Unknown	Island	promoter
148	cg03117976	chr18	11148914	11148916	PanCan	PIEZO2	Unknown	Island	promoter
149	cg23267759	chr18	11149435	11149437	Pan-BRCA	GATA6	Unknown	Island	promoter
150	cg06629130	chr18	22171373	22171375	PanCan		Intergenic	Island	CTCF binding site
151	cg23757446	chr18	47261213	47261215	Pan-BRCA	ONECUT2	TSS1500	Island	promoter
152	cg14575053	chr18	57436552	57436554	Pan-BRCA	HMSD	Unknown		CTCF binding site
153	cg23883696	chr18	63949155	63949157	PanCan	AC091138.1	Unknown	Island	promoter
154	cg26186727	chr18	72867062	72867064	PanCan	AC091138.1	Unknown	Island	promoter
155	cg18448581	chr18	72867298	72867300	PanCan	AC091138.1	Unknown	Island	promoter
156	cg24863335	chr18	72867531	72867533	PanCan	AC091138.1	Unknown	Island	promoter
157	cg03712816	chr18	72868170	72868172	PanCan	SALL3	Unknown	Island	promoter
158	cg13591058	chr19	78981156	78981158	OVC	APC2	TSS1500	Island	CTCF binding site
159	cg18612126	chr19	1445020	1445022	OVC	AC005258.1	Gene-associated	Island	CTCF binding site
160	cg11693364	chr19	2273779	2273781	OVC	ZNF69	Unknown	N_Shore	promoter
161	cg26222132	chr19	11887641	11887872	OVC	ZNF69	Unknown	N_Shore	promoter
162	cg21940042	chr19	11887641	11887872	OVC	ZNF69	Unknown	N_Shore	promoter
163	cg22319682	chr19	11887641	11887872	OVC	ZNF69	Unknown	N_Shore	promoter
164	cg03608000	chr19	11887641	11887872	OVC	ZNF69	Unknown	N_Shore	promoter
165	cg07264124	chr19	11887641	11887872	OVC	ZNF69	Unknown	N_Shore	promoter
166	cg02776664	chr19	11887641	11887872	Pan-BRCA	HOOK2	Unknown	Island	
167	cg06537894	chr19	12867809	12867893	Pan-BRCA	HOOK2	Unknown	Island	
168	cg18720973	chr19	12867809	12867893	OVC	PALM3	Unknown	S_Shore	CTCF binding site
169	cg01336912	chr19	14059153	14059208	OVC	PALM3	Unknown	S_Shore	CTCF binding site
170	cg05635754	chr19	14059153	14059208	Pan-BRCA	JAK3	Unknown	Island	promoter
171	cg09489306	chr19	17847926	17847928	Pan-BRCA	AC005622.1	Unknown	Island	CTCF binding site
172	cg06747432	chr19	43699760	43699762	Pan-BRCA	CCDC8	TSS200	Island	promoter
173	cg02583309	chr19	46413483	46413485	Pan-BRCA	AC022154.1	Unknown	N_Shore	promoter
174	cg17387989	chr19	48624069	48624254	Pan-BRCA	AC022154.1	Unknown	Island	promoter
175	cg01597066	chr19	48624069	48624254	Pan-BRCA	AC022154.1	Unknown	Island	promoter
176	cg11376305	chr19	48624069	48624254	Pan-BRCA	AC022154.1	Unknown	Island	promoter
177	cg05661282	chr19	48624069	48624254	PanCan	AC003006.1	Unknown	Island	promoter
178	cg21790626	chr19	57709001	57709149	PanCan	AC003006.1	Unknown	Island	promoter
179	cg27049766	chr19	57709001	57709149	PanCan	AC003006.1	Unknown	Island	promoter
180	cg03584535	chr19	57709001	57709149	OVC	ZNF586	Unknown	N_Shore	promoter
181	cg14038484	chr19	57769463	57769465	OVC	ZNF586	Unknown	Island	promoter
182	cg14757738	chr2	57770081	57770083	OVC	LDAH	Unknown		enhancer
183	cg14640066	chr2	20728276	20728278	OVC	RTN4	Unknown		promoter
184	cg14775296	chr2	55010284	55010286	Pan-BRCA	MEIS1	Unknown	Island	promoter
185	cg13888593	chr2	66445708	66445710	Pan-BRCA	AC005041.1	Unknown	Island	promoter

186	cg26117023	chr2	74415342	74415344	Pan-BRCA	DOK1	Unknown	Island	promoter
187	cg11270393	chr2	74554968	74554970	Pan-BRCA	ITPRIPL1	Unknown	Island	promoter
188	cg08189989	chr2	96325278	96325280	PanCan	PANTR1	Unknown	N_Shore	CTCF binding site
189	cg27404023	chr2	104842705	104842707	PanCan	AC009487.2	Unknown	Island	
190	cg04072843	chr2	161427482	161427484	OVC	KLHL23	Unknown	N_Shore	CTCF binding site
191	cg23130254	chr2	169767437	169767439	PanCan	HOXD12	Unknown	Island	
192	cg03964958	chr2	176099859	176099993	PanCan	HOXD12	Unknown	Island	
193	cg02582957	chr2	176099859	176099993	PanCan	AC013733.2	TSS200	Island	
194	cg10168149	chr2	181683227	181683229	PanCan	SATB2	Unknown	Island	
195	cg04688351	chr2	199470834	199470836	PanCan	PAX3	Unknown	N_Shore	
196	cg03462699	chr2	222289420	222289422	OVC	GAL3ST2	Gene-associated	Island	
197	cg07209686	chr20	241803798	241803800	OVC	PSMF1	Unknown	S_Shelf	
198	cg22749589	chr20	1122636	1122638	PanCan		Intergenic	N_Shore	
199	cg00960305	chr20	21521071	21521073	Pan-BRCA	LINC00261	Unknown	Island	promoter
200	cg25218716	chr20	22577658	22577660	Pan-BRCA	FOXA2	Unknown	Island	promoter
201	cg17929951	chr20	22584589	22584591	Pan-BRCA	CD40	Unknown	N_Shore	promoter
202	cg02461114	chr20	46118041	46118043	OVC	AL117379.1	Unknown	N_Shore	promoter
203	cg02133557	chr20	62929020	62929083	OVC	AL117379.1	Unknown	Island	promoter
204	cg04226366	chr20	62929020	62929083	OVC	AL117379.1	Unknown	S_Shore	promoter
205	cg00199007	chr20	62929667	62929669	Pan-BRCA	SLC17A9	Unknown	Island	promoter
206	cg04478428	chr20	62952557	62952757	Pan-BRCA	SLC17A9	Unknown	Island	promoter
207	cg01817521	chr20	62952557	62952757	Pan-BRCA	SLC17A9	Unknown	Island	promoter
208	cg17935217	chr21	62952557	62952757	Pan-BRCA	CLIC6	Unknown	Island	promoter
209	cg00495860	chr21	34669870	34669872	PanCan		Intergenic	Island	CTCF binding site
210	cg26720125	chr21	36693223	36693225	PanCan	SIM2	Unknown	Island	enhancer
211	cg04054731	chr22	36705372	36705374	Pan-BRCA	AC002472.1	Unknown	Island	promoter
212	cg27645955	chr22	21032508	21032711	Pan-BRCA	AC002472.1	Unknown	Island	promoter
213	cg24333469	chr22	21032508	21032711	Pan-BRCA	AC002472.1	Unknown	Island	promoter
214	cg05684406	chr22	21032508	21032711	Pan-BRCA	AC002472.1	Unknown	Island	promoter
215	cg23644624	chr22	21032508	21032711	Pan-BRCA	AC002472.1	Unknown	Island	promoter
216	cg13286281	chr22	21032508	21032711	Pan-BRCA	AC002472.1	Unknown	Island	promoter
217	cg01324372	chr22	21032508	21032711	Pan-BRCA	TCF20	Unknown	Island	promoter
218	cg13836638	chr22	42283570	42283572	Pan-BRCA	TCF20	Unknown	Island	promoter
219	cg22982767	chr22	42283797	42283799	OVC	AL121672.1	Unknown	N_Shelf	promoter
220	cg24961583	chr3	46058131	46058133	PanCan	EOMES	Unknown	Island	
221	cg26069719	chr3	27723917	27723919	OVC	TEX264	Unknown		CTCF binding site
222	cg12228245	chr3	51682091	51682093	OVC	AC115282.1	Unknown	S_Shore	promoter
223	cg00210994	chr3	54123529	54123531	Pan-BRCA	CMSS1	Unknown	N_Shore	promoter
224	cg15963552	chr3	99876048	99876190	Pan-BRCA	CMSS1	Unknown	N_Shore	promoter
225	cg13132965	chr3	99876048	99876190	Pan-BRCA	CMSS1	Unknown	Island	promoter
226	cg04236540	chr3	99876048	99876190	OVC	LINC01990	Unknown	N_Shore	CTCF binding site
227	cg14552401	chr3	107429959	107430001	OVC	LINC01990	Unknown	N_Shore	CTCF binding site
228	cg01208908	chr3	107429959	107430001	OVC	LINC01990	Unknown	N_Shore	CTCF binding site
229	cg22512438	chr3	107430213	107430215	PanCan	TRH	Unknown	Island	promoter
230	cg02700891	chr3	129974645	129974744	PanCan	TRH	Unknown	Island	promoter
231	cg18413131	chr3	129974645	129974744	Pan-BRCA	AC010210.1	Unknown	Island	promoter
232	cg19831706	chr3	131361852	131361854	Pan-BRCA	AC010210.1	Unknown	S_Shore	promoter
233	cg16428251	chr3	131362383	131362385	PanCan	SOX14	TSS1500	Island	
234	cg22639293	chr3	137764636	137764638	OVC	AC112504.2	Unknown	Island	

235	cg22614239	chr3	141797795	141797797	PanCan	ZIC4	Unknown	N_Shelf	
236	cg04079301	chr3	147388420	147388422	PanCan	ZIC4	Unknown	N_Shore	
237	cg18082337	chr3	147388847	147388849	PanCan	ZIC1	Unknown	Island	
238	cg12892506	chr3	147395938	147395940	PanCan	ZIC1	Unknown	Island	
239	cg00028935	chr3	147396130	147396132	PanCan	ZIC1	Unknown	Island	TF binding site
240	cg02387803	chr3	147396618	147396620	PanCan	ZIC1	Unknown	N_Shelf	
241	cg21127068	chr3	147405669	147405671	PanCan	ZIC1	Unknown	N_Shelf	
242	cg07152216	chr3	147406629	147406631	PanCan	ZIC1	Unknown	N_Shore	TF binding site
243	cg26123605	chr3	147423431	147423433	PanCan	ZIC1	Unknown	N_Shore	
244	cg05476568	chr3	147423826	147423828	OVC	ARHGEF26	Unknown	Island	promoter
245	cg03066577	chr3	154122319	154122321	OVC	SLC66A1L	Unknown	Island	CTCF binding site
246	cg12479674	chr3	157543412	157543414	OVC	AC079594.1	Unknown	Island	promoter
247	cg17078427	chr3	160450000	160450002	PanCan	AC026316.4	Unknown	Island	promoter
248	cg07091593	chr3	170419763	170419765	OVC	BCL6	Unknown	Island	CTCF binding site
249	cg17298181	chr3	187737929	187737931	OVC	BCL6	Unknown	N_Shore	promoter
250	cg03576234	chr3	187738386	187738388	OVC	BCL6	Unknown	Island	promoter
251	cg08478709	chr3	187738658	187738660	Pan-BRCA	LINC02028	Unknown	Island	CTCF binding site
252	cg08825929	chr3	194058302	194058452	Pan-BRCA	LINC02028	Unknown	Island	CTCF binding site
253	cg07907386	chr4	194058302	194058452	PanCan	PHOX2B	Unknown	Island	open_chromatin_region
254	cg14988503	chr4	41747425	41747427	Pan-BRCA	CDKL2	Unknown	Island	promoter
255	cg24432073	chr4	75630362	75630673	Pan-BRCA	CDKL2	Unknown	Island	promoter
256	cg14263942	chr4	75630362	75630673	Pan-BRCA	CDKL2	Unknown	Island	promoter
257	cg10344081	chr4	75630362	75630673	Pan-BRCA	CDKL2	Unknown	Island	promoter
258	cg21195185	chr4	75630362	75630673	Pan-BRCA	CDKL2	Unknown	Island	promoter
259	cg02466113	chr4	75630362	75630673	Pan-BRCA	CDKL2	Unknown	Island	promoter
260	cg26173997	chr4	75630362	75630673	Pan-BRCA	CDKL2	Unknown	Island	promoter
261	cg02675308	chr4	75630362	75630673	Pan-BRCA	CDKL2	Unknown	Island	promoter
262	cg23806894	chr4	75630857	75630859	PanCan	PANCR	Unknown	S_Shelf	promoter
263	cg06083330	chr4	110614371	110614373	PanCan	POU4F2	TSS1500	Island	
264	cg16887264	chr4	146640050	146640052	PanCan	POU4F2	Gene-associated	Island	enhancer
265	cg19555331	chr4	146640622	146640749	PanCan	POU4F2	Gene-associated	Island	enhancer
266	cg16509851	chr4	146640622	146640749	PanCan	AC093849.2	TSS200	Island	promoter
267	cg02348751	chr4	173509462	173509464	PanCan	TENM3	Unknown	Island	promoter
268	cg03604011	chr5	182142881	182142883	Pan-BRCA	AHRR	Unknown		
269	cg11882252	chr5	400085	400087	OVC	CMBL	Unknown	Island	promoter
270	cg05593349	chr5	10307521	10307720	OVC	CMBL	Unknown	Island	promoter
271	cg13323825	chr5	10307521	10307720	OVC	CMBL	Unknown	Island	promoter
272	cg16575104	chr5	10307521	10307720	OVC	CMBL	Unknown	S_Shore	promoter
273	cg04223424	chr5	10308133	10308135	PanCan	AC008875.2	Unknown	Island	promoter
274	cg12634932	chr5	42994673	42994675	PanCan	CCL28	Unknown	Island	promoter
275	cg10864145	chr5	43397127	43397187	PanCan	CCL28	Unknown	Island	promoter
276	cg18199554	chr5	43397127	43397187	PanCan	CCL28	Unknown	Island	promoter
277	cg13634242	chr5	43397127	43397187	Pan-BRCA	C5orf64	Unknown	Island	
278	cg15160263	chr5	61626063	61626065	PanCan		Intergenic	Island	TF binding site
279	cg10830145	chr5	77628117	77628119	Pan-BRCA	AC114971.1	Unknown	S_Shelf	promoter
280	cg18894648	chr5	88144303	88144305	PanCan	LINC00461	Unknown	S_Shore	promoter
281	cg03347590	chr5	88678628	88678630	OVC	PITX1	Unknown	N_Shore	promoter
282	cg18443359	chr5	135030471	135030473	PanCan	C5orf66	Unknown	Island	
283	cg18617005	chr5	135039002	135039004	PanCan	PCDHGA1	Unknown	Island	CTCF binding site

284	cg18507379	chr5	141407936	141407941	PanCan	PCDHGA1	Unknown	Island	CTCF binding site
285	cg14011639	chr5	141407936	141407941	PanCan	PCDHGA1	Unknown	Island	CTCF binding site
286	cg05870739	chr5	141417666	141417668	PanCan	PCDHGA1	Unknown	N_Shore	CTCF binding site
287	cg23082339	chr5	141421018	141421020	PanCan	PCDHGA1	Unknown	N_Shore	CTCF binding site
288	cg21627409	chr5	141430483	141430540	PanCan	PCDHGA1	Unknown	N_Shore	CTCF binding site
289	cg23244790	chr5	141430483	141430540	PanCan	PCDHGA1	Unknown	Island	promoter
290	cg09150633	chr5	141431534	141431536	Pan-BRCA	ADRB2	TSS200	Island	promoter
291	cg19110523	chr5	148826609	148826662	Pan-BRCA	ADRB2	TSS200	Island	promoter
292	cg15012484	chr6	148826609	148826662	Pan-BRCA	PSMG4	Unknown	Island	promoter
293	cg07109238	chr6	3232011	3232013	Pan-BRCA	H3C1	Unknown	Island	promoter
294	cg09513990	chr6	26020485	26020487	PanCan	AL021808.1	TSS200		promoter
295	cg10903903	chr6	27268042	27268044	PanCan	AL009179.2	TSS1500	Island	promoter
296	cg15845792	chr6	27680063	27680065	PanCan		Intergenic	Island	
297	cg10253847	chr6	28207667	28207669	PanCan	NKAPL	TSS200	Island	promoter
298	cg05681859	chr6	28259306	28259308	OVC	FLOT1	Unknown		CTCF binding site
299	cg04775710	chr6	30730956	30730958	OVC	HCG20	Unknown	Island	promoter
300	cg27449131	chr6	30744244	30744282	OVC	HCG20	Unknown	Island	promoter
301	cg10938374	chr6	30744244	30744282	OVC	HCG20	Unknown	Island	promoter
302	cg11485463	chr6	30744244	30744282	OVC	HSPA1A	Unknown	Island	promoter
303	cg21122656	chr6	31815462	31815769	OVC	HSPA1A	Unknown	Island	promoter
304	cg02704535	chr6	31815462	31815769	OVC	HSPA1A	Unknown	Island	promoter
305	cg19677203	chr6	31815462	31815769	OVC	HSPA1A	Unknown	Island	promoter
306	cg22847691	chr6	31815462	31815769	OVC	HSPA1A	Unknown	Island	promoter
307	cg00929855	chr6	31815462	31815769	OVC	HSPA1A	Unknown	Island	promoter
308	cg15174834	chr6	31815462	31815769	OVC	HSPA1A	Unknown	Island	promoter
309	cg12883479	chr6	31815462	31815769	OVC	HSPA1A	Unknown	Island	promoter
310	cg10598353	chr6	31815462	31815769	OVC	HSPA1A	Unknown	Island	promoter
311	cg13413286	chr6	31815462	31815769	OVC	HSPA1A	Unknown	Island	promoter
312	cg17494781	chr6	31815462	31815769	OVC	HSPA1A	Unknown	Island	promoter
313	cg18466674	chr6	31815462	31815769	OVC	HSPA1A	Unknown	Island	promoter
314	cg24509300	chr6	31815462	31815769	OVC	AL662884.3	Unknown	S_Shore	promoter
315	cg07954885	chr6	32155256	32155258	OVC	BRD2	Unknown	N_Shore	promoter
316	cg03064592	chr6	32967485	32967487	Pan-BRCA	COL21A1	Unknown	Island	CTCF binding site
317	cg15703636	chr6	56247616	56247701	Pan-BRCA	COL21A1	Unknown	S_Shore	CTCF binding site
318	cg16862641	chr6	56247616	56247701	OVC	COL9A1	Unknown	S_Shore	promoter
319	cg15368905	chr6	70283318	70283320	Pan-BRCA	HTR1B	TSS1500	Island	CTCF binding site
320	cg23424273	chr6	77462619	77462621	Pan-BRCA	HTR1B	TSS200	Island	promoter
321	cg18132916	chr6	77463509	77463511	Pan-BRCA	IRAK1BP1	Gene-associated	Island	
322	cg11833968	chr6	78910645	78910647	Pan-BRCA	IRAK1BP1	Gene-associated	Island	
323	cg21063722	chr6	78910967	78910969	PanCan	SIM1	Unknown	N_Shore	promoter
324	cg24194539	chr6	100463832	100463834	OVC	CRYBG1	Unknown	Island	
325	cg09404643	chr6	106512189	106512191	OVC	CRYBG1	Unknown	Island	
326	cg21762523	chr6	106512724	106512726	Pan-BRCA	NR2E1	Unknown	Island	enhancer
327	cg12744820	chr6	108176512	108176514	PanCan	OLIG3	TSS1500	Island	
328	cg08958294	chr6	137493822	137493824	PanCan	GRM1	Unknown	N_Shore	promoter
329	cg16842053	chr7	146028994	146028996	Pan-BRCA	CARD11	Unknown	Island	promoter
330	cg26937500	chr7	3043698	3043705	Pan-BRCA	CARD11	Unknown	Island	promoter
331	cg15285250	chr7	3043698	3043705	PanCan	NXPH1	Unknown	Island	
332	cg23536473	chr7	8442476	8442478	PanCan	AC003986.1	Unknown	Island	promoter

333	cg25691167	chr7	19144931	19144933	PanCan	AC003986.1	Unknown	Island	
334	cg24245418	chr7	19145337	19145339	PanCan	NPY	Unknown	Island	promoter
335	cg26521404	chr7	24285356	24285358	PanCan	AC004080.3	Unknown	Island	promoter
336	cg05809668	chr7	27165361	27165363	PanCan		Intergenic	Island	enhancer
337	cg16288089	chr7	35261576	35261578	PanCan	TAC1	Unknown	Island	
338	cg12914047	chr7	97732095	97732097	OVC	TRIM4	Unknown	S_Shore	promoter
339	cg13822726	chr7	99919745	99919756	OVC	TRIM4	Unknown	S_Shore	promoter
340	cg26824006	chr7	99919745	99919756	Pan-BRCA		Intergenic	Island	
341	cg03229158	chr7	122317199	122317201	OVC		Intergenic		promoter
342	cg15571405	chr7	130369578	130369580	OVC	AC016831.6	Unknown	S_Shelf	promoter
343	cg11889808	chr7	131111407	131111409	Pan-BRCA	PTPRN2	Unknown	Island	
344	cg27044841	chr8	157690755	157690757	Pan-BRCA		Intergenic	Island	
345	cg01749436	chr8	8723449	8723451	PanCan		Intergenic	Island	promoter
346	cg13021619	chr8	9898667	9898669	PanCan	AC012574.1	Unknown	Island	promoter
347	cg14428146	chr8	23706345	23706413	PanCan	AC012574.1	Unknown	Island	promoter
348	cg22747746	chr8	23706345	23706413	PanCan	AC012574.1	Unknown	Island	promoter
349	cg23290344	chr8	23706679	23706681	PanCan	AF106564.1	Unknown	Island	promoter
350	cg26205771	chr8	24913952	24913954	PanCan	NPBWR1	TSS1500	N_Shore	
351	cg05483021	chr8	52938595	52938597	OVC		Intergenic	Island	
352	cg12877723	chr8	53657107	53657109	PanCan	AC012349.1	Unknown	Island	enhancer
353	cg08851719	chr8	56445752	56445754	OVC	NCOA2	TSS1500	Island	promoter
354	cg26818625	chr8	70404394	70404396	PanCan	EYA1	Unknown	S_Shore	
355	cg14447369	chr9	71558917	71558919	OVC	CLTA	Unknown	Island	promoter
356	cg21177599	chr9	36258827	36258889	OVC	CLTA	Unknown	Island	promoter
357	cg26325209	chr9	36258827	36258889	OVC	CLTA	Unknown	Island	promoter
358	cg06912252	chr9	36258827	36258889	Pan-BRCA	PGAP4	Unknown	Island	promoter
359	cg27099274	chr9	101486643	101486645	Pan-BRCA	PGAP4	Unknown	S_Shore	promoter
360	cg06148685	chr9	101487265	101487267	Pan-BRCA	LPAR1	Unknown	Island	CTCF binding site

STROBE Checklist

Adapted from the STROBE Statement (von Elm et al., 2007) for a case-control design comparing cfDNA methylation between healthy controls (n=26, commercial biobank) and breast cancer (n=31) and ovarian cancer (n=16) cases (in-house biobank), analyzed for hypermethylated cfDNA fragments.

Section	No.	Simplified recommendation (case–control)	Applied to this study
Title & abstract	1	State it is a case-control study in the title or abstract; give a balanced summary of methods and findings.	Abstract specifies the comparison of cfDNA methylation in breast (n=31) and ovarian (n=16) cancer patients versus healthy controls (n=26)."
Introduction	2	Explain the scientific background and rationale.	Rationale needed for a non-invasive biomarker for BC/OC; cfDNA hypermethylation as a candidate signal is explained
	3	State the specific objective(s) or hypothesis.	To identify differentially hypermethylated cfDNA fragments distinguishing BC and OC cases from healthy controls."
Methods	4	Describe the study design early in the methods.	Retrospective design case-control study, two case groups (BC, OC) vs one control group has been clearly explained
	5	Describe setting, locations, and relevant dates/periods of sample collection.	Commercial biobank (controls), UK and in-house biobank (cases), University hospital, Tubingen.
	6	Give eligibility criteria for cases and controls, and how each group was selected/recruited; state rationale for choice of cases and controls.	The choice of BC and OC cases, stratified based on BRCA1 and BRCA2 status, their clinical characteristics are explained in table
	7	Define the outcome (case status) and the exposure clearly, with diagnostic criteria.	Target region -based fragment counts for cases and controls were clearly explained
	8	Describe data sources and measurement methods for each variable; note comparability of methods across groups.	cfDNA extraction, library prep, sequencing/array platform, and bioinformatics pipeline used for methylation calling are explicitly explained in methods
	9	Describe efforts to address bias.	Baseline with healthy was established, matched processing protocols, batch-correction methods, QC filtering was performed
	10	Explain the study size	Study size is explicitly mentioned as a graphical figure and in methods section
	11	Explain how quantitative variables were handled/categorized.	Thresholds of hypermethylation, fragment size thresholds are explained in detail
	12	Describe statistical methods, including handling of confounding, subgroup analyses, and missing data.	Statistical methods are explained in the Statistical analysis section

Results	13	Report numbers of individuals at each stage (screened, eligible, included, excluded) and reasons for exclusion; consider a flow diagram.	A flow diagram is included in supplementary fig1a
	14	Give participant characteristics (demographics, clinical, exposures) and note missing data.	Table 1 includes clinical characteristics of samples
	15	Report numbers/summary measures for the exposure (methylation signal) in each group.	Fraction hypermethylated fragments per group (healthy vs BC vs OC), with summary statistics (mean/median, IQrange) is reported as figure
	16	Give unadjusted and (if applicable) adjusted estimates and their precision (e.g., 95% CI, AUC).	Effect sizes for each candidate hypermethylated region/fragment sensitivity and specificity with 95% CI distinguishing cases from controls, based on Clopper and Pearson's method is reported
	17	Report any additional/subgroup/sensitivity analyses.	BC vs OC compared separately based on tumor stage, BRCA status and grading of tumor
Discussion	18	Summarize key results relative to the study objectives.	hypermethylated fragments best distinguished BC, OC from controls and are explained as figures
	19	Discuss limitations, including likely direction and magnitude of bias.	Limitations explicitly was discussed as 1. small sample sizes (26 vs 31 vs 16),2. two different biobank sources for cases vs controls (batch/technical bias), 3. Lack of independent validation cohort.
	20	Overall interpretation.	A cautious overall interpretation of the results based on healthy control to infer BC and OC samples is included
	21	Discuss generalisability (external validity) of the findings.	Need for external validation cohort and increased sample size is reported.

Reference: von Elm E, Altman DG, Egger M, Pocock SJ, Gøtzsche PC, Vandenbroucke JP; STROBE Initiative. The Strengthening the Reporting of Observational Studies in Epidemiology (STROBE) statement: guidelines for reporting observational studies.
